# Supplementary material for: Metataxonomic Mapping of the Microbial Diversity of Irish and Eastern Mediterranean Cheeses
Source: Foods. 2022 Aug 17;11(16):2483. doi: 10.3390/foods11162483 (PMC9407514; doi:10.3390/foods11162483)
Supplement: Supplementary file 1 [file foods-11-02483-s001.zip › foods-1829079-supplementary.pdf]

Supplementary material

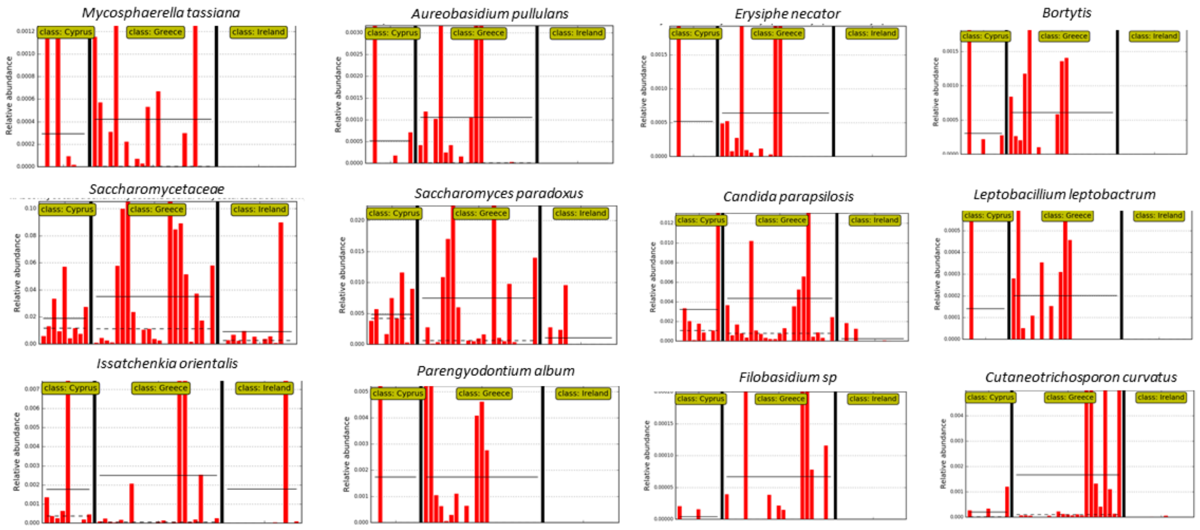

Figure S1. LEfSe analyses of taxon abundances of over-represented fungal taxa in Greek, compared to Cypriot and Irish cheeses.

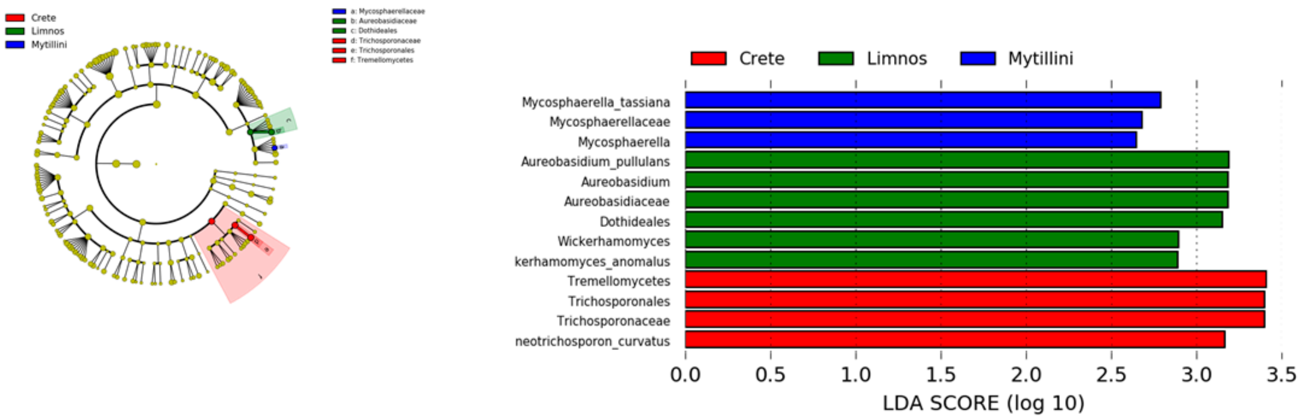

Figure S2. LEfSe analyses of taxon abundances of over-represented fungal taxa among the cheeses from the islands Crete, Limnos and Mytillini.

Table S1. Sample information, bacterial alpha diversity indexes and observed OTUs

Sample-id	Reads passing filter	Denoised reads	Shannon	Simpson	Chao	Observed OTUs

**C1**	51991	29553	4.31	0.93	64	63
**C2**	62424	46095	3.42	0.87	55	52
**C3**	108597	77111	3.33	0.86	78	63
**C4**	76361	44363	3.02	0.86	30	27
**C5**	74782	55792	3.34	0.85	69	66
**C6**	29851	16887	4.32	0.93	47	47
**C7**	43486	32370	3.45	0.87	52	52
**C8**	23297	17122	3.24	0.85	29	29
**C9**	44790	31805	3.86	0.87	74	73
**C10**	50074	37991	3.73	0.85	76	73
**GK1**	43447	28490	4.16	0.91	70	68
**GK2**	55850	36585	3.75	0.90	67	65
**GK3**	47630	32285	3.61	0.89	61	61
**GK4**	65437	45591	3.52	0.86	53	51
**GK5**	43343	33816	3.41	0.85	68	66
**GK6**	97349	67753	3.89	0.90	115	108
**GK7**	36522	25853	3.82	0.90	61	60
**GK8**	38742	26664	4.13	0.91	59	59
**GK9**	49550	37933	3.67	0.88	64	59
**GK10**	45827	37532	3.32	0.83	50	48
**GL1**	29780	23988	3.45	0.86	44	43
**GL2**	29336	19481	3.92	0.91	37	37
**GL3**	27562	17696	4.90	0.94	91	91
**GM1**	60633	35916	3.57	0.89	66	61
**GM2**	23701	19334	2.94	0.82	38	38
**GM3**	40157	29423	3.01	0.82	42	41
**GM5**	23933	17075	4.34	0.90	82	82
**GM6**	51336	33243	4.95	0.94	108	105
**GM7**	47344	31103	4.67	0.93	95	95
**GM8**	77733	56484	3.94	0.90	91	90
**GM9**	29208	21064	4.26	0.90	67	67
**GM10**	85580	64027	3.59	0.87	98	86
**I1**	25170	15637	4.03	0.92	38	38
**I2**	29384	17097	3.84	0.89	44	44
**I3**	26207	16946	4.34	0.94	38	38
**I4**	50152	33743	4.08	0.91	50	50
**I5**	63074	47793	2.37	0.78	25	24
**I6**	87038	63723	3.04	0.83	59	54
**I7**	61299	39954	3.75	0.87	95	92
**I8**	34731	24327	3.40	0.87	38	38
**I9**	28617	22715	2.48	0.78	34	33

**I10**	82182	55786	3.23	0.86	52	46
**I11**	92467	68465	2.61	0.80	28	24
**I12**	130889	82070	3.95	0.92	63	55
**I13**	81061	55472	3.55	0.86	51	45
**I14**	171827	125662	3.20	0.84	68	48
**I15**	31628	24380	2.68	0.78	53	53
**I16**	31487	15809	3.31	0.86	29	29

Table S2. Sample information, fungal alpha diversity indexes and observed OTUs

Sample-id	Reads passing filter	Denoised reads	Shannon	Simpson	Chao	Observed OTUs
**C1**	82481	63971	5.14	0.95	146	127
**C2**	69670	62531	2.65	0.70	181	145
**C3**	170315	162034	2.04	0.67	176	84
**C4**	30557	27916	4.49	0.92	96	94
**C5**	33555	26725	3.88	0.86	89	87
**C6**	23356	20764	4.71	0.92	89	89
**C7**	342772	327163	1.11	0.40	80	36
**C8**	146766	64517	5.66	0.97	165	142
**C9**	50047	48975	0.67	0.15	41	36
**C10**	29061	12493	3.86	0.87	48	48
**GK1**	67572	57274	4.18	0.85	223	175
**GK2**	76539	68417	2.22	0.61	84	76
**GK3**	28627	23997	4.04	0.89	90	87
**GK4**	160856	139602	2.02	0.43	432	256
**GK5**	71209	56749	4.61	0.93	133	123
**GK6**	51540	33419	4.53	0.88	101	98
**GK7**	82024	55020	4.48	0.89	132	109
**GK8**	173992	150490	2.01	0.69	80	46
**GK9**	46680	45952	0.57	0.15	21	18
**GK10**	41190	40597	0.30	0.07	22	21
**GL1**	50347	34742	3.14	0.63	143	139
**GL2**	62640	31720	3.55	0.73	114	112
**GL3**	57448	28619	2.14	0.45	113	106
**GM1**	44955	38554	3.40	0.77	113	103
**GM2**	178192	161084	1.52	0.38	173	95
**GM3**	73773	24773	2.39	0.64	77	75
**GM4**	29354	21145	2.29	0.66	49	47
**GM5**	55057	30757	4.68	0.89	242	223

**GM6**	57115	40176	2.68	0.71	86	81
**GM7**	82579	70367	0.80	0.16	81	67
**GM8**	32898	19354	3.18	0.67	124	124
**GM9**	90335	68752	2.07	0.49	83	76
**GM10**	28086	24844	0.53	0.12	39	38
**I1**	565986	242025	3.21	0.86	87	52
**I2**	183343	161832	0.64	0.21	19	12
**I3**	269592	214237	0.97	0.27	52	26
**I4**	355136	337284	0.97	0.33	127	47
**I5**	318122	218439	3.54	0.86	102	73
**I6**	36993	30696	2.36	0.59	52	49
**I7**	32312	26792	2.94	0.68	58	57
**I8**	27150	24086	2.53	0.60	78	72
**I9**	245590	214945	0.27	0.07	23	12
**I10**	226892	218571	0.24	0.06	63	30
**I11**	67771	51657	3.20	0.81	68	62
**I12**	95516	73684	4.94	0.95	152	120
**I13**	175839	165454	0.86	0.21	164	101
**I14**	235663	220177	1.38	0.55	76	42
**I15**	116126	85983	3.03	0.78	95	83
**I16**	73735	31980	2.56	0.77	37	36

Table S3. Comparison of the alpha microbial diversity of Cyprus, Greece and Ireland based on the Kruskal–Wallis test

Microorganism	Alpha diversity Index	Group 1	Group 2	H	p-value	q-value
**Bacteria**	Shannon	Cyprus (n=10)	Greece (n=22)	1.80	0.18	0.27
		Cyprus (n=10)	Ireland (n=16)	0.80	0.37	0.37
		Greece (n=22)	Ireland (n=16)	4.79	0.03	0.09
	Chao	Cyprus (n=10)	Greece (n=22)	1.16	0.28	0.28
		Cyprus (n=10)	Ireland (n=16)	2.51	0.11	0.17
		Greece (n=22)	Ireland (n=16)	8.85	0.00	0.01
	Simpson	Cyprus (n=10)	Greece (n=22)	1.39	0.24	0.36
		Cyprus (n=10)	Ireland (n=16)	0.47	0.49	0.49
		Greece (n=22)	Ireland (n=16)	2.74	0.10	0.29
**Fungi**	Shannon	Cyprus (n=10)	Greece (n=23)	1.77	0.18	0.27
		Cyprus (n=10)	Ireland (n=16)	4.01	0.05	0.14
		Greece (n=23)	Ireland (n=16)	1.00	0.32	0.32
	Chao	Cyprus (n=10)	Greece (n=23)	0.05	0.83	0.83
		Cyprus (n=10)	Ireland (n=16)	2.85	0.09	0.14
		Greece (n=23)	Ireland (n=16)	3.34	0.07	0.14

	Simpson	Cyprus (n=10)	Greece (n=23)	2.97	0.08	0.13
		Cyprus (n=10)	Ireland (n=16)	3.60	0.06	0.13
		Greece (n=23)	Ireland (n=16)	0.39	0.53	0.53

Table S4. Comparison of the mature cheeses' microbial beta diversity originated from Ireland, Greece and Cyprus, based on the permanova-pairwise test.

Microorganism	Unifrac distance	Group 1	Group 2	Sample size	Permutations	pseudo-F	p-value	q-value
**Bacteria**	Unweighted	Cyprus	Greece	32	999	1.31	0.159	0.159
		Cyprus	Ireland	26	999	1.71	0.048	0.072
		Greece	Ireland	38	999	2.61	0.002	0.006
	Weighted	Cyprus	Greece	32	999	1.63	0.153	0.153
		Cyprus	Ireland	26	999	12.02	0.001	0.0015
		Greece	Ireland	38	999	12.17	0.001	0.0015
**Fungi**	Unweighted	Cyprus	Greece	33	999	1.43	0.044	0.044
		Cyprus	Ireland	26	999	2.11	0.001	0.0015
		Greece	Ireland	39	999	2.89	0.001	0.0015
	Weighted	Cyprus	Greece	33	999	1.02	0.404	0.404
		Cyprus	Ireland	26	999	1.27	0.232	0.348
		Greece	Ireland	39	999	2.75	0.019	0.057

Table S5. Identified interactions between microbes in Irish cheeses in the co-occurrence network.

Cooccurance_method	Interaction Type	Interacting Microbes	weight
**dist_kullbackleibler**	**mutualExclusion**	***Brevibacterium->Candida-glaebosa***	**16.39134**
**dist_kullbackleibler**	**mutualExclusion**	***Dipodascus-australiensis->Lactobacillus-helveticus***	**20.7629**
**dist_kullbackleibler**	**mutualExclusion**	***Candida-inconspicua->Staphylococcus-equorum***	**26.23929**
**dist_kullbackleibler**	**mutualExclusion**	***Penicillium-commune->Leuconostoc***	**22.96965**
**dist_bray**	**mutualExclusion**	***Sphingomonas-yabuuchiae->Cladosporium-tenuissimum***	**1**
**dist_kullbackleibler**	**mutualExclusion**	***Methylobacterium->Candida-parapsilosis***	**17.47689**

**dist_kullbackleibler**	**mutualExclusion**	***Cladosporium-tenuissimum->Streptococcus--***	**22.02104**
**dist_kullbackleibler**	**mutualExclusion**	***Cladosporium-tenuissimum->Kluyveromyces-marxianus***	**20.93356**
**dist_kullbackleibler**	**mutualExclusion**	***Candida-zeylanoides->Turicibacter***	**19.20918**
**dist_kullbackleibler**	**mutualExclusion**	***Candida-zeylanoides->Sphingomonas-yabuuchiae***	**16.2361**
**dist_kullbackleibler**	**mutualExclusion**	***Leuconostoc-mesenteroides->Saccharomyces-paradoxus***	**17.01801**
**dist_kullbackleibler**	**mutualExclusion**	***Lactobacillus-helveticus->Methylobacterium***	**17.00658**
**dist_kullbackleibler**	**mutualExclusion**	***Candida-inconspicua->Cladosporium-tenuissimum***	**19.86373**
**dist_kullbackleibler**	**mutualExclusion**	***Candida-parapsilosis->Sphingomonas-yabuuchiae***	**18.80533**
**correl_pearson**	**copresence**	***Candida-inconspicua->Candida-glaebosa***	**0.992138**
**dist_kullbackleibler**	**mutualExclusion**	***Candida-glaebosa->Penicillium-commune***	**27.65596**
**correl_spearman**	**mutualExclusion**	***Candida-glaebosa->Staphylococcus-equorum***	**-0.65545**
**correl_pearson**	**copresence**	***Coriobacteriaceae->Sphingomonas-yabuuchiae***	**0.943858**
**dist_kullbackleibler**	**mutualExclusion**	***Candida-parapsilosis->Leuconostoc-mesenteroides***	**17.65405**
**dist_kullbackleibler**	**mutualExclusion**	***Cladosporium-tenuissimum->Leuconostoc***	**27.8182**
**correl_pearson**	**mutualExclusion**	***Lactococcus->Streptococcus--***	**-0.83263**

**dist_kullbackleibler**	**mutualExclusion**	***Lactobacillaceae-->Cladosporium-tenuissimum***	**24.57407**
**dist_kullbackleibler**	**mutualExclusion**	***Penicillium-carneum->Candida-zeylanoides***	**16.63364**
**correl_pearson**	**mutualExclusion**	***Lactococcus->Debaryomyces-hansenii***	**-0.43983**
**correl_spearman**	**mutualExclusion**	***Staphylococcus-equorum->Cladosporium-tenuissimum***	**-0.59568**
**correl_pearson**	**mutualExclusion**	***Debaryomyces-hansenii->Cladosporium-tenuissimum***	**-0.38676**
**dist_kullbackleibler**	**mutualExclusion**	***Cladosporium-tenuissimum->Penicillium-commune***	**28.62203**
**dist_kullbackleibler**	**mutualExclusion**	***Coriobacteriaceae->Cladosporium-tenuissimum***	**22.81372**
**dist_kullbackleibler**	**mutualExclusion**	***Saccharomyces-paradoxus->Sphingomonas-yabuuchiae***	**22.23392**
**correl_pearson**	**mutualExclusion**	***Lactobacillus-zeae->Lactococcus***	**-0.6045**
**dist_kullbackleibler**	**mutualExclusion**	***Candida-parapsilosis->Kluyveromyces-marxianus***	**25.23513**
**correl_pearson**	**mutualExclusion**	***Brevibacterium->Lactococcus***	**-0.72155**
**dist_bray**	**copresence**	***Candida-inconspicua->Candida-glaebosa***	**0.111504**
**dist_kullbackleibler**	**mutualExclusion**	***Leuconostoc-mesenteroides->Lactobacillus-helveticus***	**24.89518**
**correl_spearman**	**mutualExclusion**	***Kluyveromyces-marxianus->Candida-parapsilosis***	**-0.66573**
**dist_kullbackleibler**	**mutualExclusion**	***Turicibacter->Lactobacillus-helveticus***	**19.07687**

**dist_kullbackleibler**	**mutualExclusion**	***Lactobacillaceae-->Saccharomyces-paradoxus***	**17.82783**
**correl_pearson**	**mutualExclusion**	***Kluyveromyces-marxianus->Debaryomyces-hansenii***	**-0.35109**
**dist_kullbackleibler**	**mutualExclusion**	***Penicillium-commune->Penicillium-carneum***	**18.66822**
**dist_kullbackleibler**	**mutualExclusion**	***Lactobacillaceae-->Leuconostoc-mesenteroides***	**22.40701**
**correl_pearson**	**mutualExclusion**	***Candida-glaebosa->Debaryomyces-hansenii***	**-0.38242**
**dist_kullbackleibler**	**mutualExclusion**	***Sphingomonas-yabuuchiae->Candida-inconspicua***	**17.0869**
**dist_kullbackleibler**	**mutualExclusion**	***Candida-inconspicua->Turicibacter***	**19.75649**
**correl_spearman**	**mutualExclusion**	***Leuconostoc->Candida-inconspicua***	**-0.65316**
**dist_kullbackleibler**	**mutualExclusion**	***Saccharomyces-paradoxus->Kluyveromyces-marxianus***	**26.82889**
**dist_kullbackleibler**	**mutualExclusion**	***Dipodascus-australiensis->Cladosporium-tenuissimum***	**26.04503**
**dist_kullbackleibler**	**mutualExclusion**	***Candida-inconspicua->Brevibacterium***	**21.74043**
**dist_kullbackleibler**	**mutualExclusion**	***Dipodascus-australiensis->Staphylococcus-equorum***	**16.21482**
**dist_kullbackleibler**	**mutualExclusion**	***Saccharomyces-paradoxus->Methylobacterium***	**20.64541**
**dist_kullbackleibler**	**mutualExclusion**	***Candida-inconspicua->Leuconostoc-mesenteroides***	**24.82652**
**dist_kullbackleibler**	**mutualExclusion**	***Candida-inconspicua-***	**19.43514**

		>Candida-parapsilosis	
**dist_kullbackleibler**	**mutualExclusion**	***Candida-zeylanoides->Leuconostoc***	**21.17881**
**dist_kullbackleibler**	**mutualExclusion**	***Saccharomyces-paradoxus->Lactobacillus-helveticus***	**22.20736**
**dist_kullbackleibler**	**mutualExclusion**	***Penicillium-commune->Candida-parapsilosis***	**24.53845**
**dist_kullbackleibler**	**mutualExclusion**	***Brevibacterium->Candida-zeylanoides***	**16.18474**
**dist_kullbackleibler**	**mutualExclusion**	***Leuconostoc-mesenteroides->Dipodascus-australiensis***	**20.05665**
**dist_kullbackleibler**	**mutualExclusion**	***Lactobacillaceae-->Candida-inconspicua***	**16.30293**
**dist_kullbackleibler**	**mutualExclusion**	***Brevibacterium->Cladosporium-tenuissimum***	**25.87574**
**dist_kullbackleibler**	**mutualExclusion**	***Penicillium-commune->Saccharomyces-paradoxus***	**26.14964**
**correl_pearson**	**mutualExclusion**	***Debaryomyces-hansenii->Penicillium-carneum***	**-0.59451**
**correl_pearson**	**mutualExclusion**	***Candida-parapsilosis->Lactococcus***	**-0.50953**
**dist_kullbackleibler**	**mutualExclusion**	***Leuconostoc->Kluyveromyces-marxianus***	**20.38121**
**correl_spearman**	**mutualExclusion**	***Saccharomyces-paradoxus->Kluyveromyces-marxianus***	**-0.73297**
**correl_pearson**	**mutualExclusion**	***Debaryomyces-hansenii->Lactobacillus-helveticus***	**-0.54391**
**dist_kullbackleibler**	**mutualExclusion**	***Penicillium-commune->Staphylococcus-equorum***	**17.17774**

**dist_kullbackleibler**	**mutualExclusion**	***Candida-glaebosa->Staphylococcus-equorum***	**28.46422**
**dist_kullbackleibler**	**mutualExclusion**	***Lactobacillus-helveticus->Leuconostoc***	**26.00589**
**dist_kullbackleibler**	**mutualExclusion**	***Cladosporium-tenuissimum->Penicillium-carneum***	**16.79063**
**dist_kullbackleibler**	**mutualExclusion**	***Candida-parapsilosis->Staphylococcus-equorum***	**19.51852**
**dist_kullbackleibler**	**mutualExclusion**	***Leuconostoc->Candida-glaebosa***	**19.99579**
**dist_kullbackleibler**	**mutualExclusion**	***Staphylococcus-equorum->Saccharomyces-paradoxus***	**24.89657**
**correl_spearman**	**mutualExclusion**	***Candida-zeylanoides->Leuconostoc-mesenteroides***	**-0.67981**
**dist_kullbackleibler**	**mutualExclusion**	***Streptococcus--->Leuconostoc-mesenteroides***	**18.57866**
**dist_kullbackleibler**	**mutualExclusion**	***Leuconostoc->Candida-inconspicua***	**26.42399**
**dist_kullbackleibler**	**mutualExclusion**	***Candida-parapsilosis->Dipodascus-australiensis***	**16.47647**
**dist_kullbackleibler**	**mutualExclusion**	***Lactobacillaceae-->Kluyveromyces-marxianus***	**16.7719**
**dist_kullbackleibler**	**mutualExclusion**	***Kluyveromyces-marxianus->Staphylococcus-equorum***	**19.66316**
**dist_kullbackleibler**	**mutualExclusion**	***Candida-glaebosa->Coriobacteriaceae***	**16.95992**
**dist_kullbackleibler**	**mutualExclusion**	***Penicillium-commune->Leuconostoc-mesenteroides***	**16.92715**
**dist_kullbackleibler**	**mutualExclusion**	***Cladosporium-tenuissimum->Turicibacter***	**24.6891**

dist_kullbackleibler	mutualExclusion	*Brevibacterium->Kluyveromyces-marxianus*	20.24374
dist_kullbackleibler	mutualExclusion	*Leuconostoc-mesenteroides->Candida-glaebosa*	19.60258
correl_pearson	mutualExclusion	*Debaryomyces-hansenii->Candida-inconspicua*	-0.38755
dist_kullbackleibler	mutualExclusion	*Kluyveromyces-marxianus->Leuconostoc-mesenteroides*	19.46221
dist_kullbackleibler	mutualExclusion	*Leuconostoc-mesenteroides->Cladosporium-tenuissimum*	23.60545
dist_kullbackleibler	mutualExclusion	*Staphylococcus-equorum->Sphingomonas-yabuuchiae*	16.26098
dist_kullbackleibler	mutualExclusion	*Cladosporium-tenuissimum->Sphingomonas-yabuuchiae*	26.56556
dist_kullbackleibler	mutualExclusion	*Lactobacillaceae-->Lactobacillus-helveticus*	20.24025
correl_spearman	mutualExclusion	*Lactococcus->Streptococcus--*	-0.8022
dist_kullbackleibler	mutualExclusion	*Methylobacterium->Cladosporium-tenuissimum*	24.56254
dist_kullbackleibler	mutualExclusion	*Penicillium-commune->Candida-inconspicua*	16.24555
dist_kullbackleibler	mutualExclusion	*Candida-inconspicua->Methylobacterium*	18.1952
dist_kullbackleibler	mutualExclusion	*Candida-parapsilosis->Cladosporium-tenuissimum*	26.52505
dist_bray	copresence	*Sphingomonas-yabuuchiae->Coriobacteriaceae*	0.204141
dist_kullbackleibler	mutualExclusion	*Sphingomonas-yabuuchiae->Leuconostoc*	17.33768
dist_kullbackleibler	mutualExclusion	*Leuconostoc-mesenteroides-*	16.49703

		>Staphylococcus-equorum	
dist_bray	mutualExclusion	*Staphylococcus-equorum->Cladosporium-tenuissimum*	1
dist_kullbackleibler	mutualExclusion	*Staphylococcus-equorum->Candida-zeylanoides*	28.42661
dist_kullbackleibler	mutualExclusion	*Saccharomyces-paradoxus->Cladosporium-tenuissimum*	21.01705
dist_kullbackleibler	mutualExclusion	*Candida-glaebosa->Sphingomonas-yabuuchiae*	18.86257
dist_kullbackleibler	mutualExclusion	*Cladosporium-tenuissimum->Debaryomyces-hansenii*	18.1329
dist_kullbackleibler	mutualExclusion	*Saccharomyces-paradoxus->Leuconostoc*	18.68
dist_kullbackleibler	mutualExclusion	*Candida-zeylanoides->Cladosporium-tenuissimum*	18.31258
dist_kullbackleibler	mutualExclusion	*Cladosporium-tenuissimum->Staphylococcus-equorum*	29.48067
dist_kullbackleibler	mutualExclusion	*Leuconostoc-mesenteroides->Candida-zeylanoides*	21.54697
correl_spearman	mutualExclusion	*Debaryomyces-hansenii->Penicillium-carneum*	-0.66154
dist_bray	copresence	*Lactobacillus-zeae->Streptococcus--*	0.237312
dist_kullbackleibler	mutualExclusion	*Saccharomyces-paradoxus->Coriobacteriaceae*	17.10019

Table S6. Identified interactions between microbes in Greek cheeses in the co-occurrence network.

Cooccurance_method	Interaction Type	Interacting Microbes	weight

dist_kullbackleibler	mutualExclusion	*Lactobacillus-zeae->Candida-diddensiae*	17.60208
dist_kullbackleibler	mutualExclusion	*Lactobacillus-delbrueckii->Leuconostoc1*	17.57963
dist_kullbackleibler	mutualExclusion	*Acidovoraxfacilis->Staphylococcus2*	16.45945
dist_kullbackleibler	mutualExclusion	*Penicillium-carneum->Acinetobacter2*	20.76044
dist_kullbackleibler	mutualExclusion	*Chromohalobacter->Acidovoraxfacilis*	17.44057
dist_kullbackleibler	mutualExclusion	*Penicillium-carneum->Lactobacillus-hamsteri*	16.4793
dist_kullbackleibler	mutualExclusion	*Candida-diddensiae->Penicillium-carneum*	19.24153
dist_kullbackleibler	mutualExclusion	*Hanseniaspora-vineae->Candida-metapsilosis*	18.78196
dist_kullbackleibler	mutualExclusion	*Naganishia-albida->Penicillium*	16.13828
dist_bray	mutualExclusion	*Acidovoraxfacilis->Pediococcus*	1
dist_kullbackleibler	mutualExclusion	*Penicillium-commune->Acinetobacter2*	19.35716
dist_kullbackleibler	mutualExclusion	*Macrococcus->Pseudomonas2*	16.72969
dist_kullbackleibler	mutualExclusion	*Trichosporon-asahii->Wickerhamomyces-anomalus*	16.53015
dist_kullbackleibler	mutualExclusion	*Kluyveromyces-marxianus->Candida-stellata*	16.14279
dist_bray	mutualExclusion	*Weeksellaceae->Pichia-mandshurica*	1
dist_kullbackleibler	mutualExclusion	*Rhodotorula-mucilaginosa->Staphylococcus-equorum*	18.79057
dist_kullbackleibler	mutualExclusion	*Pediococcus->Acidovoraxfacilis*	17.80805
dist_kullbackleibler	mutualExclusion	*Weeksellaceae->Staphylococcus-equorum*	18.50679
correl_pearson	mutualExclusion	*Candida-stellata->Kluyveromyces-marxianus*	-0.38294
dist_kullbackleibler	mutualExclusion	*Acidovoraxfacilis->Carnobacterium*	18.3373
dist_kullbackleibler	mutualExclusion	*Malassezia-restricta->Naganishia-albida*	17.195
dist_kullbackleibler	mutualExclusion	*Rhodotorula-mucilaginosa->Leuconostoc*	16.84041
dist_kullbackleibler	mutualExclusion	*Corynebacterium-variabile->Cutaneotrichosporon-curvatus*	16.85224
dist_kullbackleibler	mutualExclusion	*Weeksellaceae->Enterobacteriaceae*	16.6838
dist_kullbackleibler	mutualExclusion	*Carnobacterium->Filobasidium-unidentified*	19.18929
dist_kullbackleibler	mutualExclusion	*Naganishia-albida->Pseudomonas2*	17.55438
dist_kullbackleibler	mutualExclusion	*Pseudomonas2->Penicillium*	18.45532

**dist_kullbackleibler**	**mutualExclusion**	***Candida-metapsilosis->Naganishia-albida***	**20.55222**
**dist_kullbackleibler**	**mutualExclusion**	***Penicillium-carneum->Saccharomyces***	**18.926**
**dist_kullbackleibler**	**mutualExclusion**	***Penicillium-carneum->Mannheimia***	**16.4806**
**dist_kullbackleibler**	**mutualExclusion**	***Kluyveromyces-marxianus->Malassezia-globosa***	**18.14068**
**dist_kullbackleibler**	**mutualExclusion**	***Filobasidium-unidentified->Naganishia-albida***	**17.0709**
**correl_spearman**	**mutualExclusion**	***Pseudomonas->Candida-diddensiae***	**-0.61863**
**dist_kullbackleibler**	**mutualExclusion**	***Penicillium-commune->Leuconostoc1***	**16.13959**
**dist_kullbackleibler**	**mutualExclusion**	***Penicillium-carneum->Hanseniaspora-vineae***	**19.59181**
**dist_kullbackleibler**	**mutualExclusion**	***Lactobacillus->Trichosporon-asahii***	**18.72234**
**dist_kullbackleibler**	**mutualExclusion**	***Penicillium-carneum->Malassezia-restricta***	**19.50515**
**dist_kullbackleibler**	**mutualExclusion**	***Naganishia->Carnobacterium***	**16.97466**
**dist_kullbackleibler**	**mutualExclusion**	***Pseudomonas2->Candida-diddensiae***	**18.08111**
**dist_kullbackleibler**	**mutualExclusion**	***Lactobacillus-zeae->Staphylococcus2***	**18.70559**
**dist_bray**	**mutualExclusion**	***Wautersiella->Rikenellaceae***	**1**
**dist_bray**	**copresence**	***Acinetobacter2->Acinetobacter***	**0.239606**
**dist_kullbackleibler**	**mutualExclusion**	***Penicillium-carneum->Penicillium-commune***	**22.74185**
**dist_kullbackleibler**	**mutualExclusion**	***Cutaneotrichosporon-curvatus->Corynebacterium***	**18.70262**
**dist_kullbackleibler**	**mutualExclusion**	***Lactobacillus-delbrueckii->Aspergillus-westerdijkiae***	**18.36909**
**dist_kullbackleibler**	**mutualExclusion**	***Saccharomyces->Kazachstania-unispora***	**19.94966**
**dist_bray**	**mutualExclusion**	***Acidovoraxfacilis->Candida-magnoliae***	**1**
**dist_bray**	**mutualExclusion**	***Malassezia-globosa->Weeksellaceae***	**1**
**dist_kullbackleibler**	**mutualExclusion**	***Saccharomyces->Malassezia-globosa***	**17.14131**
**dist_kullbackleibler**	**mutualExclusion**	***Kazachstania-unispora->Pediococcus***	**20.5288**
**correl_pearson**	**mutualExclusion**	***Lachancea-thermotolerans->Lactobacillus-helveticus***	**-0.36938**
**dist_kullbackleibler**	**mutualExclusion**	***Trichosporon-asahii->Candida-magnoliae***	**18.4733**
**dist_bray**	**copresence**	***Wautersiella->-Streptococcus***	**0.239287**
**dist_kullbackleibler**	**mutualExclusion**	***Lactobacillaceae-->Issatchenkia-orientalis***	**17.43342**
**dist_kullbackleibler**	**mutualExclusion**	***Candida-metapsilosis->Leuconostoc***	**18.23377**

dist_kullbackleibler	mutualExclusion	*Pseudomonas->Leptobacillium-leptobactrum*	16.40792
dist_kullbackleibler	mutualExclusion	*Weeksellaceae->Chromohalobacter*	19.01562
dist_bray	mutualExclusion	*Bacteroides->Carnobacterium*	1
dist_kullbackleibler	mutualExclusion	*Saccharomyces-paradoxus->Pseudomonas2*	18.73323
dist_kullbackleibler	mutualExclusion	*Corynebacterium->Lactobacillus*	20.20791
dist_kullbackleibler	mutualExclusion	*Staphylococcus2->Pediococcus*	18.43876
dist_kullbackleibler	mutualExclusion	*Lactobacillus-helveticus->Aspergillus*	16.53238
dist_kullbackleibler	mutualExclusion	*Staphylococcus2->Naganishia*	17.21588
dist_kullbackleibler	mutualExclusion	*Lachancea-thermotolerans->Chromohalobacter*	17.31051
correl_pearson	mutualExclusion	*Candida-diddensiae->Lactobacillus-helveticus*	-0.40247
dist_kullbackleibler	mutualExclusion	*Chromohalobacter->Rhodotorula-mucilaginosa*	16.58847
dist_kullbackleibler	mutualExclusion	*Naganishia-albida->Macrococcus*	19.29864
dist_kullbackleibler	mutualExclusion	*Penicillium-carneum->Aspergillus*	22.59127
dist_kullbackleibler	mutualExclusion	*Chromohalobacter->Aspergillus*	19.79868
dist_bray	mutualExclusion	*Carnobacterium->Pichia-mandshurica*	1
correl_spearman	mutualExclusion	*Lactobacillus->Corynebacterium*	-0.61662
dist_bray	mutualExclusion	*Acidovoraxfacilis->Pichia-mandshurica*	1
correl_pearson	copresence	*Issatchenkia-orientalis->Kazachstania-unispora*	0.97505
dist_kullbackleibler	mutualExclusion	*Malassezia-globosa->Pseudomonas2*	17.33391
correl_pearson	mutualExclusion	*Debaryomyces-hansenii->Rhodotorula-mucilaginosa*	-0.42445
dist_kullbackleibler	mutualExclusion	*Kazachstania-unispora->Acinetobacter2*	17.24806
dist_kullbackleibler	mutualExclusion	*Carnobacterium->Bacteroides*	17.66635
dist_kullbackleibler	mutualExclusion	*Kazachstania-unispora->Candida-stellata*	19.65216
dist_bray	mutualExclusion	*Weeksellaceae->Chromohalobacter*	1
dist_kullbackleibler	mutualExclusion	*Chromohalobacter->Penicillium-carneum*	19.04611
dist_kullbackleibler	mutualExclusion	*Leuconostoc1->Acidovoraxfacilis*	16.38708
dist_kullbackleibler	mutualExclusion	*Meyerozyma-guilliermondii->Naganishia-albida*	18.0113

dist_kullbackleibler	mutualExclusion	*Bifidobacteriaceae->Lactobacillaceae-*	16.28158
correl_pearson	mutualExclusion	*Debaryomyces-hansenii->Cutaneotrichosporon-curvatus*	-0.37161
dist_kullbackleibler	mutualExclusion	*Penicillium-carneum->Lactobacillus-helveticus*	16.39912
dist_kullbackleibler	mutualExclusion	*Candida-metapsilosis->Malassezia-globosa*	17.69067
dist_kullbackleibler	mutualExclusion	*Carnobacterium->Rikenellaceae*	17.33956
dist_kullbackleibler	mutualExclusion	*Penicillium-carneum->Candida-metapsilosis*	17.80391
correl_pearson	mutualExclusion	*Debaryomyces-hansenii->Penicillium*	-0.46248
correl_pearson	mutualExclusion	*Debaryomyces-hansenii->Issatchenkia-orientalis*	-0.37606
correl_spearman	mutualExclusion	*Candida-magnoliae->Candida-inconspicua*	-0.66273
dist_bray	mutualExclusion	*Leptobacillium-leptobactrum->Pseudomonas2*	1
dist_kullbackleibler	mutualExclusion	*Candida-magnoliae->Rhodotorula-mucilaginosa*	16.57245
dist_kullbackleibler	mutualExclusion	*Wickerhamomyces-anomalus->Staphylococcus-equorum*	17.22854
dist_kullbackleibler	mutualExclusion	*Staphylococcus2->Weeksellaceae*	17.29379
dist_bray	mutualExclusion	*Lactobacillus-hamsteri->Leuconostoc1*	1
correl_pearson	mutualExclusion	*Lactobacillus-helveticus->Leptobacillium-leptobactrum*	-0.34528
dist_bray	mutualExclusion	*Penicillium->Weeksellaceae*	1
dist_kullbackleibler	mutualExclusion	*Lachancea-thermotolerans->Lactobacillus-helveticus*	17.02243
dist_kullbackleibler	mutualExclusion	*Lachancea-thermotolerans->Kazachstania-unispora*	20.43082
dist_kullbackleibler	mutualExclusion	*Wickerhamomyces-anomalus->Chromohalobacter*	17.98307
dist_kullbackleibler	mutualExclusion	*Trichosporon-asahii->Leptobacillium-leptobactrum*	20.91411
dist_kullbackleibler	mutualExclusion	*Leptobacillium-leptobactrum->Candida-inconspicua*	16.437
correl_pearson	mutualExclusion	*Acidovoraxfacilis->Debaryomyces-hansenii*	-0.34895
dist_kullbackleibler	mutualExclusion	*Weeksellaceae->Malassezia-globosa*	18.4552
dist_kullbackleibler	mutualExclusion	*Candida-metapsilosis->Leptobacillium-leptobactrum*	17.86381
correl_spearman	mutualExclusion	*Bifidobacteriaceae->Lactobacillaceae-*	-0.6207
dist_kullbackleibler	mutualExclusion	*Acinetobacter2->Weeksellaceae*	16.7188
correl_pearson	mutualExclusion	*Penicillium-carneum->Streptococcus--*	-0.44121
dist_kullbackleibler	mutualExclusion	*Kazachstania-unispora->Leuconostoc*	17.34169
correl_pearson	mutualExclusion	*Lactococcus->Streptococcus--*	-0.82384

**dist_kullbackleibler**	**mutualExclusion**	***Carnobacterium->Weeksellaceae***	**19.91263**
**dist_kullbackleibler**	**mutualExclusion**	***Pseudomonas2->Penicillium-carneum***	**21.60356**
**dist_bray**	**mutualExclusion**	***Pseudomonas2->Pichia-mandshurica***	**1**
**dist_bray**	**mutualExclusion**	***Kazachstania-unispora->Aspergillus-westerdijkiae***	**1**
**dist_kullbackleibler**	**mutualExclusion**	***Issatchenkia-orientalis->Staphylococcus-equorum***	**20.60905**
**dist_kullbackleibler**	**mutualExclusion**	***Issatchenkia-orientalis->Pseudomonas2***	**18.39911**
**dist_kullbackleibler**	**mutualExclusion**	***Candida-parapsilosis->Pseudomonas2***	**18.0076**
**dist_kullbackleibler**	**mutualExclusion**	***Malassezia-globosa->Trichosporon-asahii***	**17.80631**
**dist_kullbackleibler**	**mutualExclusion**	***Carnobacterium->Penicillium***	**16.26311**
**dist_kullbackleibler**	**mutualExclusion**	***Lactobacillus-delbrueckii->Penicillium-carneum***	**23.13523**
**dist_kullbackleibler**	**mutualExclusion**	***Candida-metapsilosis->Penicillium***	**16.58841**
**dist_kullbackleibler**	**mutualExclusion**	***Corynebacterium->Lactobacillus-zeae***	**16.90995**
**dist_kullbackleibler**	**mutualExclusion**	***Aspergillus-westerdijkiae->Candida-metapsilosis***	**19.11598**
**dist_kullbackleibler**	**mutualExclusion**	***Kazachstania-unispora->Pichia-mandshurica***	**18.03237**
**dist_kullbackleibler**	**mutualExclusion**	***Chromohalobacter->Candida-metapsilosis***	**19.70967**
**correl_pearson**	**mutualExclusion**	***Chryseobacterium->Rikenellaceae***	**-0.34323**
**dist_kullbackleibler**	**mutualExclusion**	***Kazachstania-unispora->Rikenellaceae***	**18.29199**
**dist_kullbackleibler**	**mutualExclusion**	***Kluyveromyces-marxianus->Hanseniaspora-vineae***	**18.53355**
**correl_spearman**	**mutualExclusion**	***Candida-inconspicua->Debaryomyces-hansenii***	**-0.59569**
**dist_kullbackleibler**	**mutualExclusion**	***Penicillium-carneum->Lachancea-thermotolerans***	**21.37962**
**dist_kullbackleibler**	**mutualExclusion**	***Weeksellaceae->Hanseniaspora-vineae***	**18.35306**
**dist_kullbackleibler**	**mutualExclusion**	***Penicillium-commune->Saccharomyces-paradoxus***	**17.63572**
**dist_kullbackleibler**	**mutualExclusion**	***Filobasidium-unidentified->Leuconostoc1***	**17.32491**
**correl_spearman**	**mutualExclusion**	***Bifidobacteriaceae->Rikenellaceae***	**-0.66234**
**dist_kullbackleibler**	**mutualExclusion**	***Carnobacterium->Leptobacillium-leptobactrum***	**17.54667**
**dist_kullbackleibler**	**mutualExclusion**	***Naganishia-albida->Kocuria***	**18.13802**
**dist_kullbackleibler**	**mutualExclusion**	***Wickerhamomyces-anomalus->Pseudomonas2***	**16.31855**

**dist_kullbackleibler**	**mutualExclusion**	***Trichosporon-asahii->Aspergillus***	**17.79905**
**correl_spearman**	**mutualExclusion**	***Naganishia->Lactococcus***	**-0.57286**
**dist_kullbackleibler**	**mutualExclusion**	***Penicillium-commune->-Streptococcus***	**21.65655**
**dist_kullbackleibler**	**mutualExclusion**	***Candida-magnoliae->Weeksellaceae***	**16.90347**
**dist_kullbackleibler**	**mutualExclusion**	***Chromohalobacter->Issatchenkia-orientalis***	**19.86359**
**dist_kullbackleibler**	**mutualExclusion**	***Rhodotorula-mucilaginosa->Staphylococcus2***	**19.11884**
**correl_spearman**	**mutualExclusion**	***Cutaneotrichosporon-curvatus->Malassezia-restricta***	**-0.60803**
**dist_kullbackleibler**	**mutualExclusion**	***Meyerozyma-guilliermondii->Lactobacillus-zeae***	**18.49381**
**dist_kullbackleibler**	**mutualExclusion**	***Issatchenkia-orientalis->Staphylococcus2***	**19.57034**
**dist_kullbackleibler**	**mutualExclusion**	***Penicillium-carneum->Candida-stellata***	**20.97346**
**dist_kullbackleibler**	**mutualExclusion**	***Weeksellaceae->Penicillium***	**19.69711**
**dist_kullbackleibler**	**mutualExclusion**	***Pichia-mandshurica->Lactobacillus-zeae***	**16.42509**
**dist_kullbackleibler**	**mutualExclusion**	***Weeksellaceae->Macrococcus***	**17.06393**
**dist_kullbackleibler**	**mutualExclusion**	***Lactobacillus-hamsteri->Staphylococcus2***	**19.8437**
**dist_bray**	**mutualExclusion**	***Carnobacterium->Acidovoraxfacilis***	**1**
**dist_kullbackleibler**	**mutualExclusion**	***Mycosphaerella-tassiana->Pediococcus***	**16.61641**
**dist_kullbackleibler**	**mutualExclusion**	***Lactobacillus->Enterobacteriaceae***	**19.15991**
**dist_kullbackleibler**	**mutualExclusion**	***Penicillium-carneum->Lactobacillaceae-***	**17.16983**
**dist_kullbackleibler**	**mutualExclusion**	***Aspergillus-westerdijkiae->Issatchenkia-orientalis***	**16.62265**
**dist_kullbackleibler**	**mutualExclusion**	***Streptococcus-equi->Penicillium-carneum***	**20.83516**
**dist_kullbackleibler**	**mutualExclusion**	***Kazachstania-unispora->Staphylococcus2***	**20.69069**
**dist_kullbackleibler**	**mutualExclusion**	***Naganishia-albida->Acinetobacter2***	**20.08051**
**dist_kullbackleibler**	**mutualExclusion**	***Lactobacillus->Corynebacterium-variabile***	**19.85037**
**dist_kullbackleibler**	**mutualExclusion**	***Naganishia-albida->Corynebacterium***	**17.9756**
**dist_kullbackleibler**	**mutualExclusion**	***Penicillium-commune->Penicillium***	**16.68207**
**dist_kullbackleibler**	**mutualExclusion**	***Corynebacterium-variabile->Penicillium-commune***	**17.10293**
**dist_bray**	**mutualExclusion**	***Candida-diddensiae->Lactobacillus-hamsteri***	**1**

dist_kullbackleibler	mutualExclusion	*Penicillium-commune->Macrococcus*	18.21398
dist_kullbackleibler	mutualExclusion	*Staphylococcus-equorum->Lactobacillus*	21.49589
correl_spearman	mutualExclusion	*Streptococcus--->Lactococcus*	-0.63185
dist_kullbackleibler	mutualExclusion	*Chromohalobacter->Kazachstania-unispora*	20.35449
correl_spearman	mutualExclusion	*Malassezia-restricta->Lactobacillus-helveticus*	-0.58743
dist_kullbackleibler	mutualExclusion	*Kazachstania-unispora->Naganishia-albida*	21.78819
dist_kullbackleibler	mutualExclusion	*Aspergillus-westerdijkiae->Kazachstania-unispora*	21.59875
dist_kullbackleibler	mutualExclusion	*Penicillium-carneum->Saccharomyces-paradoxus*	16.38804
dist_kullbackleibler	mutualExclusion	*Candida-magnoliae->Penicillium-carneum*	18.0553
dist_kullbackleibler	mutualExclusion	*Penicillium-carneum->Wickerhamomyces-anomalus*	20.17794
dist_kullbackleibler	mutualExclusion	*Aspergillus-westerdijkiae->Naganishia-albida*	19.03954
dist_kullbackleibler	mutualExclusion	*Chromohalobacter->Candida-stellata*	16.91178
correl_pearson	mutualExclusion	*Lactobacillus->Streptococcus--*	-0.50557
dist_bray	mutualExclusion	*Lactobacillus-hamsteri->Pichia-mandshurica*	1
correl_pearson	mutualExclusion	*Lactobacillaceae-->Kocuria*	-0.35299
dist_kullbackleibler	mutualExclusion	*Staphylococcus-equorum->Penicillium-carneum*	22.81738
dist_kullbackleibler	mutualExclusion	*Cladosporium-tenuissimum->Pseudomonas2*	17.91035
dist_kullbackleibler	mutualExclusion	*Corynebacterium-variabile->Naganishia-albida*	18.42674
dist_kullbackleibler	mutualExclusion	*Malassezia-restricta->Penicillium-commune*	16.66665
dist_kullbackleibler	mutualExclusion	*Naganishia-albida->Streptococcus-equi*	17.05213
correl_spearman	mutualExclusion	*Lactobacillus-zeae->Corynebacterium-variabile*	-0.63241
dist_kullbackleibler	mutualExclusion	*-Streptococcus->Pediococcus*	17.21055
dist_kullbackleibler	mutualExclusion	*Candida-magnoliae->Candida-metapsilosis*	17.05999
dist_kullbackleibler	mutualExclusion	*Penicillium-carneum->Naganishia*	16.78756
correl_pearson	mutualExclusion	*Kluyveromyces-marxianus->Candida-magnoliae*	-0.37881
dist_kullbackleibler	mutualExclusion	*Acinetobacter->Penicillium-carneum*	19.85771
dist_kullbackleibler	mutualExclusion	*Saccharomyces->Penicillium-commune*	20.77853

**correl_pearson**	**copresence**	***Carnobacterium->-Streptococcus***	**0.947239**
**correl_spearman**	**mutualExclusion**	***Saccharomyces-paradoxus->Lactobacillus-hamsteri***	**-0.58694**
**correl_pearson**	**copresence**	***Acinetobacter->Acinetobacter2***	**0.946654**
**dist_kullbackleibler**	**mutualExclusion**	***Issatchenkia-orientalis->Malassezia-globosa***	**16.73758**
**dist_kullbackleibler**	**mutualExclusion**	***Staphylococcus2->Penicillium-commune***	**20.47179**
**dist_kullbackleibler**	**mutualExclusion**	***Staphylococcus-equorum->Leptobacillium-leptobactrum***	**17.40169**
**dist_bray**	**mutualExclusion**	***Rikenellaceae->Carnobacterium***	**1**
**dist_kullbackleibler**	**mutualExclusion**	***Kluyveromyces-marxianus->Candida-magnoliae***	**16.1673**
**dist_kullbackleibler**	**mutualExclusion**	***Cladosporium-tenuissimum->Penicillium-commune***	**18.3629**
**dist_kullbackleibler**	**mutualExclusion**	***Candida-diddensiae->Lactobacillus-helveticus***	**16.70994**
**dist_kullbackleibler**	**mutualExclusion**	***Corynebacterium-variabile->Penicillium-carneum***	**16.18924**
**dist_kullbackleibler**	**mutualExclusion**	***Lachancea-thermotolerans->Trichosporon-asahii***	**20.30503**
**dist_kullbackleibler**	**mutualExclusion**	***Lactobacillus-zeae->Corynebacterium-variabile***	**20.94249**
**correl_spearman**	**mutualExclusion**	***Leptobacillium-leptobactrum->Lactobacillus-helveticus***	**-0.62894**
**dist_kullbackleibler**	**mutualExclusion**	***Candida-metapsilosis->Staphylococcus-equorum***	**17.91508**
**dist_kullbackleibler**	**mutualExclusion**	***Leuconostoc1->Pediococcus***	**16.7408**
**dist_kullbackleibler**	**mutualExclusion**	***Kazachstania-unispora->Cladosporium-tenuissimum***	**17.71904**
**dist_bray**	**mutualExclusion**	***Weeksellaceae->Lachancea-thermotolerans***	**1**
**correl_spearman**	**mutualExclusion**	***Lactobacillus-helveticus->Lactococcus***	**-0.63704**
**dist_kullbackleibler**	**mutualExclusion**	***Saccharomyces-paradoxus->Lactobacillus-hamsteri***	**18.66102**
**dist_bray**	**mutualExclusion**	***Lactobacillus-hamsteri->Lachancea-thermotolerans***	**1**
**dist_kullbackleibler**	**mutualExclusion**	***Candida-diddensiae->Kazachstania-unispora***	**19.44491**
**dist_kullbackleibler**	**mutualExclusion**	***Carnobacterium->Saccharomyces***	**19.03165**
**dist_kullbackleibler**	**mutualExclusion**	***Penicillium-commune->Pichia-mandshurica***	**17.82366**
**dist_kullbackleibler**	**mutualExclusion**	***Penicillium-commune->Staphylococcus-equorum***	**20.84369**
**dist_kullbackleibler**	**mutualExclusion**	***Leptobacillium-leptobactrum->Penicillium-commune***	**16.8437**
**dist_kullbackleibler**	**mutualExclusion**	***Naganishia->Aspergillus***	**17.40121**

**dist_kullbackleibler**	**mutualExclusion**	***Weeksellaceae->Leuconostoc1***	**17.96195**
**dist_bray**	**mutualExclusion**	***Naganishia->Leuconostoc1***	**1**
**dist_kullbackleibler**	**mutualExclusion**	***Naganishia-albida->Hanseniaspora-vineae***	**18.40143**
**dist_kullbackleibler**	**mutualExclusion**	***Penicillium-carneum->Malassezia-globosa***	**22.32357**
**dist_bray**	**mutualExclusion**	***Saccharomyces->Carnobacterium***	**1**
**dist_bray**	**copresence**	***Corynebacterium->Corynebacterium-variabile***	**0.200646**
**dist_kullbackleibler**	**mutualExclusion**	***Naganishia-albida->Trichosporon-asahii***	**22.81673**
**correl_pearson**	**mutualExclusion**	***Debaryomyces-hansenii->Kazachstania-unispora***	**-0.33397**
**dist_kullbackleibler**	**mutualExclusion**	***Cutaneotrichosporon-curvatus->Staphylococcus-equorum***	**16.30534**
**dist_kullbackleibler**	**mutualExclusion**	***Enterobacteriaceae->Penicillium-commune***	**20.00549**
**dist_kullbackleibler**	**mutualExclusion**	***Pediococcus->Candida-metapsilosis***	**17.49021**
**correl_pearson**	**mutualExclusion**	***Saccharomyces-paradoxus->Debaryomyces-hansenii***	**-0.35368**
**dist_kullbackleibler**	**mutualExclusion**	***Weeksellaceae->Saccharomyces***	**16.89911**
**correl_spearman**	**mutualExclusion**	***Staphylococcus-equorum->Lactobacillus***	**-0.64129**
**correl_spearman**	**mutualExclusion**	***Kluyveromyces-marxianus->Debaryomyces-hansenii***	**-0.63885**
**correl_spearman**	**mutualExclusion**	***Staphylococcus2->Lactobacillus***	**-0.71604**
**dist_kullbackleibler**	**mutualExclusion**	***Meyerozyma-guilliermondii->Weeksellaceae***	**16.75088**
**dist_kullbackleibler**	**mutualExclusion**	***Chromohalobacter->Pediococcus***	**19.36169**
**dist_kullbackleibler**	**mutualExclusion**	***Pediococcus->Meyerozyma-guilliermondii***	**17.57046**
**dist_kullbackleibler**	**mutualExclusion**	***Carnobacterium->Lactobacillus-delbrueckii***	**22.6725**
**dist_bray**	**mutualExclusion**	***Weeksellaceae->Candida-magnoliae***	**1**
**dist_bray**	**mutualExclusion**	***Acidovoraxfacilis->Rikenellaceae***	**1**
**dist_kullbackleibler**	**mutualExclusion**	***Weeksellaceae->Pichia-mandshurica***	**17.06984**
**dist_kullbackleibler**	**mutualExclusion**	***Kazachstania-unispora->Hanseniaspora-vineae***	**20.27787**
**dist_kullbackleibler**	**mutualExclusion**	***Candida-stellata->Trichosporon-asahii***	**18.52485**
**dist_kullbackleibler**	**mutualExclusion**	***Trichosporon-asahii->Pediococcus***	**16.30807**
**dist_kullbackleibler**	**mutualExclusion**	***Pediococcus->Candida-diddensiae***	**16.81888**
**dist_kullbackleibler**	**mutualExclusion**	***Pseudomonas2->Mycosphaerella-tassiana***	**18.92008**

**dist_kullbackleibler**	**mutualExclusion**	***Kazachstania-unispora->Leptobacillium-leptobactrum***	**20.57323**
**dist_kullbackleibler**	**mutualExclusion**	***Lactobacillus-zeae->Chromohalobacter***	**21.89207**
**dist_kullbackleibler**	**mutualExclusion**	***Chromohalobacter->Leptobacillium-leptobactrum***	**18.74054**
**dist_kullbackleibler**	**mutualExclusion**	***Malassezia-restricta->Kazachstania-unispora***	**20.33381**
**dist_kullbackleibler**	**mutualExclusion**	***Kazachstania-unispora->Carnobacterium***	**19.51136**
**dist_kullbackleibler**	**mutualExclusion**	***Lactobacillus-delbrueckii->Staphylococcus2***	**16.17589**
**dist_kullbackleibler**	**mutualExclusion**	***Staphylococcus2->Naganishia-albida***	**16.7438**
**dist_kullbackleibler**	**mutualExclusion**	***Corynebacterium->Kazachstania-unispora***	**19.63219**
**dist_kullbackleibler**	**mutualExclusion**	***Wautersiella->Weeksellaceae***	**16.44468**
**dist_kullbackleibler**	**mutualExclusion**	***Candida-metapsilosis->Carnobacterium***	**16.26855**
**dist_kullbackleibler**	**mutualExclusion**	***Kazachstania-unispora->Pseudomonas2***	**17.90229**
**dist_kullbackleibler**	**mutualExclusion**	***Pediococcus->Wautersiella***	**16.15493**
**dist_kullbackleibler**	**mutualExclusion**	***Aspergillus->Penicillium-commune***	**16.34224**
**dist_kullbackleibler**	**mutualExclusion**	***-Streptococcus->Penicillium-carneum***	**16.75817**
**dist_kullbackleibler**	**mutualExclusion**	***Candida-metapsilosis->Malassezia-restricta***	**17.95577**
**dist_kullbackleibler**	**mutualExclusion**	***Filobasidium-unidentified->Malassezia-globosa***	**16.80591**
**dist_kullbackleibler**	**mutualExclusion**	***Saccharomyces->Lactobacillus-helveticus***	**17.09974**
**dist_kullbackleibler**	**mutualExclusion**	***Candida-diddensiae->Pseudomonas***	**16.21589**
**dist_kullbackleibler**	**mutualExclusion**	***Malassezia-restricta->Issatchenkia-orientalis***	**16.43953**
**dist_kullbackleibler**	**mutualExclusion**	***Penicillium-commune->Candida-diddensiae***	**17.63554**
**dist_kullbackleibler**	**mutualExclusion**	***Wautersiella->Trichosporon-asahii***	**17.42672**
**dist_kullbackleibler**	**mutualExclusion**	***Rhodotorula-mucilaginosa->Aspergillus***	**17.44174**
**correl_spearman**	**mutualExclusion**	***Pseudomonas->Lactococcus***	**-0.66838**
**dist_kullbackleibler**	**mutualExclusion**	***Kazachstania-unispora->Trichosporon-asahii***	**17.18075**
**dist_kullbackleibler**	**mutualExclusion**	***Lactobacillus-delbrueckii->Kazachstania-unispora***	**17.0266**
**dist_kullbackleibler**	**mutualExclusion**	***Aspergillus->Naganishia-albida***	**16.34682**

dist_kullbackleibler	mutualExclusion	*Malassezia-globosa->Kazachstania-unispora*	16.99517
correl_pearson	mutualExclusion	*Debaryomyces-hansenii->Candida-inconspicua*	-0.5158
dist_kullbackleibler	mutualExclusion	*Candida-diddensiae->Candida-metapsilosis*	19.58427
dist_kullbackleibler	mutualExclusion	*Saccharomyces->Hanseniaspora-vineae*	16.70933
dist_kullbackleibler	mutualExclusion	*Pediococcus->Corynebacterium-variabile*	19.22175
dist_kullbackleibler	mutualExclusion	*Lachancea-thermotolerans->Weeksellaceae*	18.59296
dist_bray	mutualExclusion	*Bacteroides->Leuconostoc1*	1
dist_kullbackleibler	mutualExclusion	*Carnobacterium->Penicillium-commune*	22.64877
dist_kullbackleibler	mutualExclusion	*Carnobacterium->Pichia-mandshurica*	18.15093
correl_spearman	mutualExclusion	*Lactobacillaceae-->Lactobacillus-helveticus*	-0.60739
dist_kullbackleibler	mutualExclusion	*Lachancea-thermotolerans->Lactobacillus-hamsteri*	16.37979
dist_kullbackleibler	mutualExclusion	*Hanseniaspora-vineae->Trichosporon-asahii*	20.97191
dist_kullbackleibler	mutualExclusion	*Pediococcus->Naganishia-albida*	18.621
correl_pearson	mutualExclusion	*Debaryomyces-hansenii->Saccharomyces*	-0.37615
correl_spearman	mutualExclusion	*Pseudomonas->Pichia-mandshurica*	-0.60241
dist_kullbackleibler	mutualExclusion	*Lactobacillus->Chromohalobacter*	21.43877
correl_pearson	mutualExclusion	*Malassezia-globosa->Streptococcus--*	-0.41093
dist_kullbackleibler	mutualExclusion	*Candida-metapsilosis->Saccharomyces*	17.64962
dist_bray	mutualExclusion	*Aspergillus-westerdijkiae->Weeksellaceae*	1
dist_kullbackleibler	mutualExclusion	*Wickerhamomyces-anomalus->Pediococcus*	16.90223
dist_kullbackleibler	mutualExclusion	*Leptobacillium-leptobactrum->Pseudomonas2*	19.51139
dist_bray	mutualExclusion	*Naganishia-albida->Naganishia*	1
dist_kullbackleibler	mutualExclusion	*Filobasidium-unidentified->Hanseniaspora-vineae*	16.2362
dist_bray	mutualExclusion	*Carnobacterium->Rhodotorula-mucilaginosa*	1
dist_kullbackleibler	mutualExclusion	*Naganishia->Lachancea-thermotolerans*	16.98818
dist_kullbackleibler	mutualExclusion	*Lactobacillaceae-->Rhodotorula-mucilaginosa*	16.83777
dist_kullbackleibler	mutualExclusion	*Lactobacillus-delbrueckii->Chromohalobacter*	21.17871

dist_kullbackleibler	mutualExclusion	*Penicillium-carneum->Penicillium*	20.24447
dist_kullbackleibler	mutualExclusion	*Staphylococcus2->Penicillium-carneum*	22.88194
dist_kullbackleibler	mutualExclusion	*Penicillium-carneum->Macrococcus*	19.09503
dist_kullbackleibler	mutualExclusion	*Lactobacillus-delbrueckii->Trichosporon-asahii*	16.15455
dist_kullbackleibler	mutualExclusion	*Candida-metapsilosis->Aspergillus*	18.76253
dist_kullbackleibler	mutualExclusion	*Naganishia->Leuconostoc1*	17.14907
dist_kullbackleibler	mutualExclusion	*Leptobacillium-leptobactrum->Lactobacillus-zeae*	16.34506
dist_kullbackleibler	mutualExclusion	*Saccharomyces->Lactobacillus-zeae*	18.25443
dist_kullbackleibler	mutualExclusion	*Kluyveromyces-marxianus->Chromohalobacter*	17.36165
dist_kullbackleibler	mutualExclusion	*Saccharomyces-paradoxus->Naganishia-albida*	21.57989
dist_kullbackleibler	mutualExclusion	*Issatchenkia-orientalis->Carnobacterium*	17.01752
dist_kullbackleibler	mutualExclusion	*Filobasidium-unidentified->Pediococcus*	18.04847
dist_kullbackleibler	mutualExclusion	*Macrococcus->Lactobacillus*	17.00907
dist_kullbackleibler	mutualExclusion	*Carnobacterium->Rhodotorula-mucilaginosa*	21.32257
dist_kullbackleibler	mutualExclusion	*Trichosporon-asahii->Carnobacterium*	20.57508
dist_kullbackleibler	mutualExclusion	*Pseudomonas2->Leuconostoc*	16.87461
dist_kullbackleibler	mutualExclusion	*Penicillium-commune->Wautersiella*	20.31222
dist_kullbackleibler	mutualExclusion	*Lactobacillus-delbrueckii->Candida-inconspicua*	16.30691
dist_kullbackleibler	mutualExclusion	*Enhydrobacter->Penicillium-carneum*	18.70255
dist_kullbackleibler	mutualExclusion	*Candida-diddensiae->Lactobacillus-hamsteri*	16.95671
dist_kullbackleibler	mutualExclusion	*Pseudomonas2->Lachancea-thermotolerans*	16.74934
dist_bray	mutualExclusion	*Saccharomyces->Lactobacillus-hamsteri*	1
dist_kullbackleibler	mutualExclusion	*Cutaneotrichosporon-curvatus->Staphylococcus2*	18.29967
dist_kullbackleibler	mutualExclusion	*Saccharomyces-paradoxus->Chromohalobacter*	17.13338
dist_kullbackleibler	mutualExclusion	*Leptobacillium-leptobactrum->Penicillium-carneum*	23.82958
dist_kullbackleibler	mutualExclusion	*Hanseniaspora-vineae->Lactobacillus-helveticus*	18.47455

**dist_kullbackleibler**	**mutualExclusion**	***Leptobacillium-leptobactrum->Kluyveromyces-marxianus***	**17.96458**
**dist_kullbackleibler**	**mutualExclusion**	***Issatchenkia-orientalis->Pediococcus***	**19.86632**
**dist_kullbackleibler**	**mutualExclusion**	***Penicillium-commune->Meyerozyma-guilliermondii***	**21.67924**
**dist_kullbackleibler**	**mutualExclusion**	***Leuconostoc1->Penicillium-carneum***	**19.7233**
**dist_kullbackleibler**	**mutualExclusion**	***Penicillium-carneum->Trichosporon-asahii***	**18.97597**
**dist_kullbackleibler**	**mutualExclusion**	***Staphylococcus-equorum->Saccharomyces-paradoxus***	**17.0162**
**dist_kullbackleibler**	**mutualExclusion**	***Rikenellaceae->Lactobacillus-delbrueckii***	**17.68309**
**dist_kullbackleibler**	**mutualExclusion**	***Enterobacteriaceae->Penicillium-carneum***	**22.43718**
**correl_pearson**	**mutualExclusion**	***Debaryomyces-hansenii->Candida-zeylanoides***	**-0.40438**
**dist_kullbackleibler**	**mutualExclusion**	***Kazachstania-unispora->Candida-magnoliae***	**17.49466**
**dist_kullbackleibler**	**mutualExclusion**	***Naganishia-albida->Naganishia***	**19.08097**
**dist_kullbackleibler**	**mutualExclusion**	***Carnobacterium->Lactobacillus-zeae***	**18.50368**
**dist_kullbackleibler**	**mutualExclusion**	***Staphylococcus-equorum->Kazachstania-unispora***	**22.23723**
**dist_kullbackleibler**	**mutualExclusion**	***-Streptococcus->Pseudomonas2***	**16.4933**
**dist_bray**	**mutualExclusion**	***Lactobacillus-hamsteri->Hanseniaspora-vineae***	**1**
**dist_kullbackleibler**	**mutualExclusion**	***Candida-stellata->Candida-metapsilosis***	**19.31166**
**dist_bray**	**copresence**	***Enterobacteriaceae->Acinetobacter2***	**0.24609**
**dist_kullbackleibler**	**mutualExclusion**	***Pediococcus->Penicillium***	**16.56327**
**dist_kullbackleibler**	**mutualExclusion**	***Pediococcus->Macrococcus***	**18.36855**
**dist_kullbackleibler**	**mutualExclusion**	***Macrococcus->Penicillium***	**16.7672**
**dist_kullbackleibler**	**mutualExclusion**	***Lactobacillus->-Streptococcus***	**18.54255**
**correl_pearson**	**mutualExclusion**	***Leuconostoc1->Streptococcus--***	**-0.46136**
**dist_bray**	**mutualExclusion**	***Weeksellaceae->Hanseniaspora-vineae***	**1**
**dist_kullbackleibler**	**mutualExclusion**	***Pichia-mandshurica->Candida-metapsilosis***	**18.39227**
**dist_kullbackleibler**	**mutualExclusion**	***Lactobacillus-zeae->Staphylococcus-equorum***	**21.23802**
**correl_pearson**	**copresence**	***Meyerozyma-guilliermondii->Candida-parapsilosis***	**0.955807**
**dist_kullbackleibler**	**mutualExclusion**	***Naganishia-albida->Leuconostoc***	**18.68767**
**dist_kullbackleibler**	**mutualExclusion**	***Saccharomyces->Pseudomonas2***	**16.82115**

dist_kullbackleibler	mutualExclusion	*Saccharomyces->Pediococcus*	17.06933
dist_kullbackleibler	mutualExclusion	*Pediococcus->Penicillium-carneum*	22.25122
correl_pearson	mutualExclusion	*Lactococcus->Lactobacillus-helveticus*	-0.39078
dist_kullbackleibler	mutualExclusion	*Candida-metapsilosis->Lachancea-thermotolerans*	17.24923
dist_kullbackleibler	mutualExclusion	*Staphylococcus-equorum->Lactobacillus-delbrueckii*	21.40974
dist_kullbackleibler	mutualExclusion	*Leuconostoc->Issatchenkia-orientalis*	18.97771
dist_kullbackleibler	mutualExclusion	*Penicillium-commune->Candida-magnoliae*	17.48415
dist_kullbackleibler	mutualExclusion	*Meyerozyma-guilliermondii->Pseudomonas2*	18.52218
dist_kullbackleibler	mutualExclusion	*Weeksellaceae->Aspergillus-westerdijkiae*	19.56511
dist_kullbackleibler	mutualExclusion	*Corynebacterium-variabile->Lactobacillaceae-*	16.80438
correl_pearson	mutualExclusion	*Kluyveromyces-marxianus->Debaryomyces-hansenii*	-0.61866
dist_kullbackleibler	mutualExclusion	*Naganishia-albida->Staphylococcus-equorum*	17.50224
dist_kullbackleibler	mutualExclusion	*Candida-metapsilosis->Mycosphaerella-tassiana*	17.7596
dist_bray	mutualExclusion	*Chromohalobacter->Acidovoraxfacilis*	1
dist_kullbackleibler	mutualExclusion	*Hanseniaspora-vineae->Naganishia*	17.13598
dist_kullbackleibler	mutualExclusion	*Penicillium-carneum->Pichia-mandshurica*	16.75669
dist_kullbackleibler	mutualExclusion	*Rikenellaceae->Issatchenkia-orientalis*	16.24811
dist_kullbackleibler	mutualExclusion	*Penicillium-carneum->Pseudomonas*	18.44395
dist_kullbackleibler	mutualExclusion	*Penicillium-carneum->Cladosporium-tenuissimum*	16.19402
dist_kullbackleibler	mutualExclusion	*Leuconostoc1->Lactobacillus*	18.09467
dist_kullbackleibler	mutualExclusion	*Pediococcus->Rhodotorula-mucilaginosa*	19.55234
dist_kullbackleibler	mutualExclusion	*Penicillium-commune->Corynebacterium*	16.38442
dist_kullbackleibler	mutualExclusion	*Hanseniaspora-vineae->Lactobacillus-hamsteri*	16.14357
dist_kullbackleibler	mutualExclusion	*Lactobacillaceae-->Kazachstania-unispora*	17.10426
dist_bray	mutualExclusion	*Carnobacterium->Weeksellaceae*	1
dist_kullbackleibler	mutualExclusion	*Penicillium-carneum->Wautersiella*	16.26507
dist_kullbackleibler	mutualExclusion	*Aspergillus->Lactobacillus-hamsteri*	16.26115

dist_kullbackleibler	mutualExclusion	*Kazachstania-unispora->Mycosphaerella-tassiana*	21.4632
correl_spearman	mutualExclusion	*Malassezia-restricta->Candida-inconspicua*	-0.60278
correl_spearman	mutualExclusion	*Lachancea-thermotolerans->Lactobacillus-helveticus*	-0.58617
correl_pearson	mutualExclusion	*Malassezia-restricta->Lactobacillus-helveticus*	-0.41996
correl_spearman	mutualExclusion	*Lactobacillus-helveticus->Candida-diddensiae*	-0.5832
dist_bray	mutualExclusion	*Aspergillus-westerdijkiae->Lactobacillus-hamsteri*	1
dist_kullbackleibler	mutualExclusion	*Chromohalobacter->Penicillium-commune*	22.59487
dist_kullbackleibler	mutualExclusion	*Issatchenkia-orientalis->Candida-diddensiae*	16.14096
dist_kullbackleibler	mutualExclusion	*Lactobacillus-helveticus->Leptobacillium-leptobactrum*	19.75498
dist_kullbackleibler	mutualExclusion	*Carnobacterium->Penicillium-carneum*	17.09581
dist_kullbackleibler	mutualExclusion	*Pseudomonas2->Kluyveromyces-marxianus*	17.00997
dist_bray	mutualExclusion	*Kazachstania-unispora->Rikenellaceae*	1
dist_kullbackleibler	mutualExclusion	*Lachancea-thermotolerans->Penicillium-commune*	17.41355
dist_kullbackleibler	mutualExclusion	*Kazachstania-unispora->Corynebacterium-variabile*	16.37535
dist_kullbackleibler	mutualExclusion	*Chromohalobacter->Cladosporium-tenuissimum*	16.50627
dist_kullbackleibler	mutualExclusion	*Aspergillus-westerdijkiae->Lactobacillus-hamsteri*	17.35347
dist_kullbackleibler	mutualExclusion	*Naganishia-albida->Rhodotorula-mucilaginosa*	20.72453
dist_kullbackleibler	mutualExclusion	*Staphylococcus2->Lactobacillus*	24.23028
dist_kullbackleibler	mutualExclusion	*Lactobacillus->Carnobacterium*	22.26671
dist_kullbackleibler	mutualExclusion	*Naganishia-albida->Carnobacterium*	17.93828
dist_kullbackleibler	mutualExclusion	*Lactobacillus-helveticus->Malassezia-globosa*	17.07592
dist_kullbackleibler	mutualExclusion	*-Streptococcus->Lactobacillus-hamsteri*	16.71327
correl_pearson	mutualExclusion	*Filobasidium-unidentified->Debaryomyces-hansenii*	-0.4235
dist_kullbackleibler	mutualExclusion	*Pseudomonas2->Pichia-mandshurica*	17.02532
dist_kullbackleibler	mutualExclusion	*Naganishia-albida->Enterobacteriaceae*	20.35047
dist_kullbackleibler	mutualExclusion	*Hanseniaspora-vineae->Chromohalobacter*	16.40324

dist_bray	mutualExclusion	*Weeksellaceae->Leuconostoc1*	1
dist_bray	copresence	*Kazachstania-unispora->Issatchenkia-orientalis*	0.160059
dist_kullbackleibler	mutualExclusion	*Aspergillus->Kazachstania-unispora*	21.97412
dist_bray	copresence	*Mannheimia->Wickerhamomyces-anomalus*	0.246099
dist_bray	mutualExclusion	*Acidovoraxfacilis->Leuconostoc1*	1
dist_kullbackleibler	mutualExclusion	*Lactobacillus-delbrueckii->Issatchenkia-orientalis*	16.24979
dist_kullbackleibler	mutualExclusion	*Corynebacterium->Pseudomonas2*	17.60639
dist_kullbackleibler	mutualExclusion	*Malassezia-globosa->Naganishia-albida*	18.06731
dist_kullbackleibler	mutualExclusion	*Aspergillus-westerdijkiae->Penicillium-carneum*	17.5164
dist_kullbackleibler	mutualExclusion	*Torulaspora-delbrueckii->Acinetobacter-johnsonii*	17.96496
correl_pearson	copresence	*Torulaspora-delbrueckii->Leuconostoc-mesenteroides*	0.943984
dist_kullbackleibler	mutualExclusion	*Torulaspora-delbrueckii->Kurthiaibsonii*	18.31669
dist_kullbackleibler	mutualExclusion	*Torulaspora-delbrueckii->Kluyveromyces-lactis*	22.79399
dist_kullbackleibler	mutualExclusion	*Torulaspora-delbrueckii->Acinetobacter-uillouiae*	17.03131
dist_kullbackleibler	mutualExclusion	*Torulaspora-delbrueckii->Klebsiella*	19.38422
dist_kullbackleibler	mutualExclusion	*Botryosphaeria-dothidea->Kluyveromyces-lactis*	17.33751
correl_spearman	copresence	*Erysiphe-necator->Stemphylium*	0.973064
correl_spearman	copresence	*Erysiphe-necator->Penicillium-spinulosum*	0.826282
dist_kullbackleibler	copresence	*Erysiphe-necator->Penicillium-spinulosum*	0.65008
dist_bray	copresence	*Erysiphe-necator->Aureobasidium-pullulans*	0.090866
correl_spearman	copresence	*Erysiphe-necator->Aureobasidium-pullulans*	0.869771
correl_pearson	copresence	*Erysiphe-necator->Aureobasidium-pullulans*	0.99377
dist_kullbackleibler	copresence	*Erysiphe-necator->Aureobasidium-pullulans*	0.235607
correl_spearman	copresence	*Erysiphe-necator->Hanseniaspora*	0.848026
correl_spearman	copresence	*Parengyodontium-album->Erysiphe-necator*	0.889562
correl_spearman	copresence	*Parengyodontium-album->Botrytis*	0.853007

**dist_kullbackleibler**	**mutualExclusion**	***Parengyodontium-album->Kluyveromyces-lactis***	**19.22835**
**dist_kullbackleibler**	**mutualExclusion**	***Parengyodontium-album->Yamadazyma-triangularis***	**19.35171**
**correl_spearman**	**copresence**	***Hanseniaspora-nectarophila->Erysiphe-necator***	**0.887772**
**correl_spearman**	**copresence**	***Hanseniaspora-nectarophila->Stemphylium***	**0.917559**
**dist_bray**	**copresence**	***Hanseniaspora-nectarophila->Stemphylium***	**0.167005**
**dist_kullbackleibler**	**mutualExclusion**	***Hanseniaspora-nectarophila->Yamadazyma-triangularis***	**16.87606**
**correl_spearman**	**copresence**	***Hanseniaspora-nectarophila->Hanseniaspora***	**0.920634**
**dist_bray**	**copresence**	***Hanseniaspora-nectarophila->Hanseniaspora***	**0.162916**
**correl_pearson**	**copresence**	***Acinetobacter-johnsonii->Botrytis***	**0.950821**
**correl_pearson**	**copresence**	***Acinetobacter-johnsonii->Acinetobacter-uillouiae***	**0.993118**
**dist_bray**	**copresence**	***Acinetobacter-johnsonii->Acinetobacter-uillouiae***	**0.125256**
**correl_pearson**	**copresence**	***Acinetobacter-johnsonii->Acinetobacter-rhizosphaerae***	**0.971956**
**correl_pearson**	**copresence**	***Acinetobacter-johnsonii->Klebsiella***	**0.95524**
**dist_bray**	**copresence**	***Acinetobacter-johnsonii->Klebsiella***	**0.209788**
**dist_kullbackleibler**	**mutualExclusion**	***Acinetobacter-johnsonii->Lactobacillus-brevis***	**18.78356**
**dist_kullbackleibler**	**mutualExclusion**	***Enhydrobacter-->Yamadazyma-triangularis***	**16.8936**
**dist_kullbackleibler**	**copresence**	***Stemphylium->Hanseniaspora-nectarophila***	**0.58111**
**dist_kullbackleibler**	**copresence**	***Stemphylium->Erysiphe-necator***	**0.740776**
**correl_spearman**	**copresence**	***Stemphylium->Parengyodontium-album***	**0.890909**
**dist_kullbackleibler**	**copresence**	***Stemphylium->Botrytis***	**0.569752**
**dist_kullbackleibler**	**copresence**	***Stemphylium->Penicillium-spinulosum***	**0.485649**
**dist_bray**	**copresence**	***Stemphylium->Penicillium-spinulosum***	**0.237687**
**correl_spearman**	**copresence**	***Stemphylium->Penicillium-spinulosum***	**0.889538**
**dist_kullbackleibler**	**mutualExclusion**	***Stemphylium->Kluyveromyces-lactis***	**17.63836**
**correl_spearman**	**copresence**	***Stemphylium->Alternaria-alternata***	**0.839461**
**dist_kullbackleibler**	**copresence**	***Stemphylium->Hanseniaspora***	**0.305526**

**correl_spearman**	**copresence**	***Stemphylium->Hanseniaspora***	**0.89546 9**
**dist_bray**	**copresence**	***Stemphylium->Hanseniaspora***	**0.16862 3**
**correl_pearson**	**copresence**	***Sporobolomyces-roseus->Kurthiaibsonii***	**0.95550 3**
**dist_kullbackleibler**	**mutualExclusion**	***Sporobolomyces-roseus->Kluyveromyces-lactis***	**18.8005 9**
**correl_pearson**	**copresence**	***Sporobolomyces-roseus->Aureobasidium-pullulans***	**0.95267**
**dist_kullbackleibler**	**mutualExclusion**	***Sporobolomyces-roseus->Yamadazyma-triangularis***	**16.7385 2**
**dist_kullbackleibler**	**mutualExclusion**	***Leuconostoc-mesenteroides->Erysiphe-necator***	**16.143**
**dist_bray**	**copresence**	***Botrytis->Sporobolomyces-roseus***	**0.22075 1**
**dist_kullbackleibler**	**copresence**	***Botrytis->Erysiphe-necator***	**0.39963 2**
**correl_pearson**	**copresence**	***Botrytis->Erysiphe-necator***	**0.96970 2**
**correl_spearman**	**copresence**	***Botrytis->Stemphylium***	**0.94432 7**
**correl_spearman**	**copresence**	***Botrytis->Erysiphe-necator***	**0.92218 9**
**correl_pearson**	**copresence**	***Botrytis->Sporobolomyces-roseus***	**0.95881 9**
**dist_bray**	**copresence**	***Botrytis->Erysiphe-necator***	**0.16858 8**
**correl_spearman**	**copresence**	***Botrytis->Hanseniaspora-nectarophila***	**0.88543 8**
**correl_pearson**	**copresence**	***Botrytis->Kurthiaibsonii***	**0.96171 1**
**dist_kullbackleibler**	**copresence**	***Botrytis->Penicillium-spinulosum***	**0.90674 8**
**dist_kullbackleibler**	**mutualExclusion**	***Botrytis->Kluyveromyces-lactis***	**21.3711 6**
**correl_pearson**	**copresence**	***Botrytis->Alternaria-metachromatica***	**0.96459 9**
**correl_pearson**	**copresence**	***Botrytis->Acinetobacter-rhizosphaerae***	**0.95392 3**
**correl_pearson**	**copresence**	***Botrytis->Aureobasidium-pullulans***	**0.98156 5**
**correl_pearson**	**copresence**	***Kurthiaibsonii->Acinetobacter-johnsonii***	**0.95536 2**
**dist_kullbackleibler**	**mutualExclusion**	***Kurthiaibsonii->Leuconostoc-mesenteroides***	**19.6103 4**
**dist_kullbackleibler**	**mutualExclusion**	***Kurthiaibsonii->Candida-atlantica***	**20.7584 3**
**dist_bray**	**mutualExclusion**	***Kurthiaibsonii->Candida-atlantica***	**1**
**dist_bray**	**copresence**	***Kurthiaibsonii->Acinetobacter-rhizosphaerae***	**0.20064 7**

correl_pearson	copresence	*Kurthiaibsonii->Acinetobacter-rhizosphaerae*	0.984584
correl_spearman	copresence	*Penicillium-spinulosum->Hanseniaspora-nectarophila*	0.911763
correl_pearson	copresence	*Penicillium-spinulosum->Stemphylium*	0.953583
dist_kullbackleibler	mutualExclusion	*Penicillium-spinulosum->Candida-atlantica*	16.81801
dist_kullbackleibler	copresence	*Penicillium-spinulosum->Aureobasidium-pullulans*	0.364781
dist_kullbackleibler	mutualExclusion	*Kluyveromyces-lactis->Kurthiaibsonii*	18.22601
dist_kullbackleibler	mutualExclusion	*Kluyveromyces-lactis->Enhydrobacter-*	19.01204
dist_kullbackleibler	mutualExclusion	*Kluyveromyces-lactis->Acinetobacter-johnsonii*	17.79139
dist_kullbackleibler	mutualExclusion	*Kluyveromyces-lactis->Penicillium-spinulosum*	18.12039
dist_kullbackleibler	mutualExclusion	*Kluyveromyces-lactis->Leuconostoc-mesenteroides*	16.87275
dist_kullbackleibler	mutualExclusion	*Kluyveromyces-lactis->Hanseniaspora-nectarophila*	18.08635
dist_kullbackleibler	mutualExclusion	*Kluyveromyces-lactis->Erysiphe-necator*	21.50525
dist_kullbackleibler	mutualExclusion	*Kluyveromyces-lactis->Macrococcus1*	17.61795
dist_kullbackleibler	mutualExclusion	*Kluyveromyces-lactis->Candida-atlantica*	17.38196
dist_kullbackleibler	mutualExclusion	*Kluyveromyces-lactis->Cladosporium-sphaerospermum*	17.10939
dist_kullbackleibler	mutualExclusion	*Kluyveromyces-lactis->Alternaria-alternata*	17.32588
dist_kullbackleibler	mutualExclusion	*Kluyveromyces-lactis->Acinetobacter-rhizosphaerae*	16.84369
dist_kullbackleibler	mutualExclusion	*Kluyveromyces-lactis->Aureobasidium-pullulans*	19.85431
dist_kullbackleibler	mutualExclusion	*Kluyveromyces-lactis->Enterococcus*	17.42921
dist_bray	mutualExclusion	*Candida-atlantica->Enhydrobacter-*	1
dist_kullbackleibler	mutualExclusion	*Candida-atlantica->Sporobolomyces-roseus*	18.37922
dist_kullbackleibler	mutualExclusion	*Candida-atlantica->Enhydrobacter-*	19.25312
dist_kullbackleibler	mutualExclusion	*Candida-atlantica->Torulaspora-delbrueckii*	16.93072
dist_kullbackleibler	mutualExclusion	*Cladosporium-sphaerospermum->Torulaspora-delbrueckii*	16.59538
dist_kullbackleibler	mutualExclusion	*Cladosporium-sphaerospermum->Yamadazyma-triangularis*	16.17036
dist_kullbackleibler	mutualExclusion	*Cladosporium-sphaerospermum->Lactobacillus-brevis*	17.58263

**correl_spearman**	**mutualExclusion**	***Alternaria-metachromatica->Kluyveromyces-lactis***	**-0.63339**
**dist_bray**	**copresence**	***Alternaria-metachromatica->Botrytis***	**0.20676**
**dist_kullbackleibler**	**mutualExclusion**	***Alternaria-metachromatica->Kluyveromyces-lactis***	**21.1718**
**dist_bray**	**copresence**	***Alternaria-metachromatica->Alternaria-alternata***	**0.218552**
**dist_bray**	**copresence**	***Alternaria-metachromatica->Aureobasidium-pullulans***	**0.215239**
**correl_pearson**	**copresence**	***Acinetobacter-uillouiae->Kurthiaibsonii***	**0.967726**
**dist_kullbackleibler**	**mutualExclusion**	***Acinetobacter-uillouiae->Kluyveromyces-lactis***	**17.41642**
**correl_pearson**	**copresence**	***Acinetobacter-uillouiae->Botrytis***	**0.948819**
**dist_kullbackleibler**	**copresence**	***Acinetobacter-uillouiae->Acinetobacter-johnsonii***	**0.829049**
**dist_kullbackleibler**	**copresence**	***Acinetobacter-uillouiae->Acinetobacter-rhizosphaerae***	**0.982917**
**dist_bray**	**copresence**	***Acinetobacter-uillouiae->Klebsiella***	**0.243971**
**correl_pearson**	**copresence**	***Alternaria-alternata->Alternaria-metachromatica***	**0.946406**
**correl_spearman**	**copresence**	***Alternaria-alternata->Erysiphe-necator***	**0.832871**
**dist_bray**	**copresence**	***Alternaria-alternata->Aureobasidium-pullulans***	**0.213615**
**dist_kullbackleibler**	**mutualExclusion**	***Acinetobacter-rhizosphaerae->Candida-atlantica***	**16.61872**
**dist_bray**	**copresence**	***Acinetobacter-rhizosphaerae->Acinetobacter-uillouiae***	**0.136676**
**correl_pearson**	**copresence**	***Acinetobacter-rhizosphaerae->Acinetobacter-uillouiae***	**0.984445**
**dist_kullbackleibler**	**mutualExclusion**	***Acinetobacter-rhizosphaerae->Torulaspora-delbrueckii***	**18.2243**
**dist_kullbackleibler**	**mutualExclusion**	***Acinetobacter-rhizosphaerae->Leuconostoc-mesenteroides***	**17.13681**
**dist_bray**	**copresence**	***Acinetobacter-rhizosphaerae->Acinetobacter-johnsonii***	**0.249005**
**correl_pearson**	**copresence**	***Acinetobacter-rhizosphaerae->Sporobolomyces-roseus***	**0.951375**
**dist_kullbackleibler**	**mutualExclusion**	***Acinetobacter-rhizosphaerae->Lactobacillus-brevis***	**18.5078**
**dist_kullbackleibler**	**copresence**	***Aureobasidium-pullulans->Botrytis***	**0.340127**
**correl_spearman**	**copresence**	***Aureobasidium-pullulans->Botrytis***	**0.846846**
**dist_bray**	**copresence**	***Aureobasidium-pullulans->Botrytis***	**0.150303**
**dist_bray**	**copresence**	***Aureobasidium-pullulans->Sporobolomyces-roseus***	**0.241903**

**dist_kullbackleibler**	**copresence**	***Aureobasidium-pullulans->Stemphylium***	**0.317546**
**correl_spearman**	**copresence**	***Aureobasidium-pullulans->Penicillium-spinulosum***	**0.917473**
**dist_bray**	**copresence**	***Aureobasidium-pullulans->Penicillium-spinulosum***	**0.217727**
**correl_spearman**	**copresence**	***Aureobasidium-pullulans->Stemphylium***	**0.915895**
**dist_bray**	**copresence**	***Aureobasidium-pullulans->Stemphylium***	**0.24614**
**dist_kullbackleibler**	**mutualExclusion**	***Aureobasidium-pullulans->Yamadazyma-triangularis***	**17.96845**
**dist_kullbackleibler**	**mutualExclusion**	***Yamadazyma-triangularis->Penicillium-spinulosum***	**18.32854**
**dist_kullbackleibler**	**mutualExclusion**	***Yamadazyma-triangularis->Kurthiaibsonii***	**16.27829**
**dist_kullbackleibler**	**mutualExclusion**	***Yamadazyma-triangularis->Kluyveromyces-lactis***	**16.96664**
**dist_kullbackleibler**	**mutualExclusion**	***Yamadazyma-triangularis->Torulaspora-delbrueckii***	**18.34156**
**dist_kullbackleibler**	**mutualExclusion**	***Yamadazyma-triangularis->Lactobacillus-brevis***	**21.14898**
**dist_kullbackleibler**	**mutualExclusion**	***Yamadazyma-triangularis->Hanseniaspora***	**17.49149**
**correl_spearman**	**mutualExclusion**	***Enterococcus->Kluyveromyces-lactis***	**-0.61405**
**dist_kullbackleibler**	**mutualExclusion**	***Klebsiella->Kluyveromyces-lactis***	**16.80921**
**dist_kullbackleibler**	**mutualExclusion**	***Lactobacillus-brevis->Klebsiella***	**20.38368**
**dist_kullbackleibler**	**mutualExclusion**	***Lactobacillus-brevis->Kluyveromyces-lactis***	**19.67692**
**dist_kullbackleibler**	**mutualExclusion**	***Lactobacillus-brevis->Candida-atlantica***	**17.98235**
**dist_kullbackleibler**	**mutualExclusion**	***Lactobacillus-brevis->Kurthiaibsonii***	**20.93294**
**dist_kullbackleibler**	**mutualExclusion**	***Lactobacillus-brevis->Acinetobacter-uillouiae***	**18.70881**
**correl_pearson**	**copresence**	***Lactobacillus-brevis->Torulaspora-delbrueckii***	**0.943844**
**dist_kullbackleibler**	**copresence**	***Hanseniaspora->Aureobasidium-pullulans***	**0.986668**
**dist_kullbackleibler**	**copresence**	***Hanseniaspora->Hanseniaspora-nectarophila***	**0.23723**
**dist_kullbackleibler**	**mutualExclusion**	***Hanseniaspora->Kluyveromyces-lactis***	**16.33022**
**correl_spearman**	**copresence**	***Hanseniaspora->Penicillium-spinulosum***	**0.864603**
**correl_spearman**	**copresence**	***Hanseniaspora->Botrytis***	**0.910478**
**correl_spearman**	**copresence**	***Erysiphe-necator->Leptobacillium-leptobactrum***	**0.853007**

dist_kullbackleibler	mutualExclusion	*Kazachstania-unispora->Yamadazyma-triangularis*	17.40583
dist_kullbackleibler	mutualExclusion	*Penicillium->Leuconostoc-mesenteroides*	17.77416
dist_kullbackleibler	mutualExclusion	*Penicillium-carneum->Penicillium-spinulosum*	21.77435
dist_kullbackleibler	mutualExclusion	*Pediococcus->Acinetobacter-rhizosphaerae*	18.1291
correl_pearson	copresence	*Acinetobacter-rhizosphaerae->Acinetobacter2*	0.947452
dist_kullbackleibler	mutualExclusion	*Cladosporium-sphaerospermum->Pseudomonas2*	16.66381
dist_kullbackleibler	mutualExclusion	*Candida-stellata->Yamadazyma-triangularis*	18.16542
dist_kullbackleibler	mutualExclusion	*Issatchenkia-orientalis->Lactobacillus-brevis*	21.80469
dist_kullbackleibler	mutualExclusion	*Mannheimia->Torulaspora-delbrueckii*	16.80432
dist_kullbackleibler	mutualExclusion	*Hanseniaspora->Lactobacillus-hamsteri*	18.55847
dist_kullbackleibler	mutualExclusion	*Lactobacillus-hamsteri->Candida-atlantica*	16.83056
dist_kullbackleibler	mutualExclusion	*Parengyodontium-album->Staphylococcus-equorum*	18.98734
dist_kullbackleibler	mutualExclusion	*Penicillium-carneum->Aureobasidium-pullulans*	20.73004
dist_kullbackleibler	mutualExclusion	*Kurthiaibsonii->Leuconostoc1*	16.47381
dist_kullbackleibler	mutualExclusion	*Pseudomonas2->Acinetobacter-uillouiae*	16.32709
dist_kullbackleibler	mutualExclusion	*Penicillium->Candida-atlantica*	17.92873
correl_spearman	mutualExclusion	*Lactobacillus-brevis->Kluyveromyces-marxianus*	-0.61354
dist_kullbackleibler	mutualExclusion	*Hanseniaspora-nectarophila->Candida-inconspicua*	16.57295
dist_bray	copresence	*Acinetobacter2->Acinetobacter-uillouiae*	0.238689
dist_kullbackleibler	mutualExclusion	*Enhydrobacter-->Pediococcus*	19.59576
dist_kullbackleibler	mutualExclusion	*Lactobacillus-hamsteri->Macrococcus1*	17.36819
dist_kullbackleibler	mutualExclusion	*Pseudomonas2->Hanseniaspora-nectarophila*	18.80316
dist_kullbackleibler	mutualExclusion	*Meyerozyma-guilliermondii->Leuconostoc-mesenteroides*	16.17884
dist_kullbackleibler	mutualExclusion	*Chromohalobacter->Parengyodontium-album*	20.1229
dist_kullbackleibler	mutualExclusion	*Issatchenkia-orientalis->Enhydrobacter-*	17.4864
dist_kullbackleibler	mutualExclusion	*Chromohalobacter->Enterococcus*	19.34917

**dist_kullbackleibler**	**mutualExclusion**	***Pseudomonas2->Stemphylium***	**18.47045**
**dist_kullbackleibler**	**mutualExclusion**	***Cutaneotrichosporon-curvatus->Hanseniaspora-nectarophila***	**19.00626**
**dist_kullbackleibler**	**mutualExclusion**	***Macrococcus->Kurthiaibsonii***	**17.63475**
**dist_kullbackleibler**	**mutualExclusion**	***Bifidobacteriaceae->Lactobacillus-brevis***	**18.21046**
**dist_kullbackleibler**	**mutualExclusion**	***Carnobacterium->Sporobolomyces-roseus***	**20.73456**
**dist_kullbackleibler**	**mutualExclusion**	***Candida-atlantica->Chromohalobacter***	**16.6192**
**dist_kullbackleibler**	**mutualExclusion**	***Klebsiella->Trichosporon-asahii***	**17.95521**
**dist_kullbackleibler**	**mutualExclusion**	***Lactobacillus-zeae->Candida-atlantica***	**16.33129**
**dist_kullbackleibler**	**mutualExclusion**	***Penicillium-commune->Stemphylium***	**19.50583**
**dist_kullbackleibler**	**mutualExclusion**	***Enhydrobacter-->Penicillium-carneum***	**24.288**
**dist_kullbackleibler**	**mutualExclusion**	***Streptococcus-equi->Acinetobacter-uillouiae***	**16.59146**
**dist_kullbackleibler**	**mutualExclusion**	***Lactobacillus-hamsteri->Parengyodontium-album***	**16.95098**
**dist_kullbackleibler**	**mutualExclusion**	***Weeksellaceae->Sporobolomyces-roseus***	**19.82269**
**dist_kullbackleibler**	**mutualExclusion**	***Botrytis->Pediococcus***	**18.1656**
**dist_kullbackleibler**	**mutualExclusion**	***Penicillium-carneum->Stemphylium***	**22.84705**
**dist_kullbackleibler**	**mutualExclusion**	***Penicillium-commune->Penicillium-spinulosum***	**18.88431**
**dist_kullbackleibler**	**mutualExclusion**	***Cladosporium-sphaerospermum->Trichosporon-asahii***	**18.83232**
**dist_kullbackleibler**	**mutualExclusion**	***Lactobacillus-helveticus->Parengyodontium-album***	**18.08292**
**dist_bray**	**copresence**	***Hanseniaspora->Lachancea-thermotolerans***	**0.235499**
**dist_kullbackleibler**	**mutualExclusion**	***Hanseniaspora->Cutaneotrichosporon-curvatus***	**16.8976**
**correl_spearman**	**mutualExclusion**	***Cutaneotrichosporon-curvatus->Alternaria-metachromatica***	**-0.65244**
**dist_kullbackleibler**	**mutualExclusion**	***Acinetobacter-uillouiae->Weeksellaceae***	**17.32037**
**dist_kullbackleibler**	**mutualExclusion**	***Candida-atlantica->Saccharomyces***	**17.60155**
**dist_kullbackleibler**	**mutualExclusion**	***Macrococcus1->Candida-metapsilosis***	**16.47919**
**dist_kullbackleibler**	**mutualExclusion**	***Hanseniaspora-nectarophila->Penicillium-carneum***	**24.03757**
**dist_kullbackleibler**	**mutualExclusion**	***Penicillium-commune->Kurthiaibsonii***	**19.06528**
**dist_kullbackleibler**	**mutualExclusion**	***Corynebacterium-variabile->Lactobacillus-brevis***	**20.1933**

dist_kullbackleibler	mutualExclusion	*Enhydrobacter-->Kazachstania-unispora*	18.74494
dist_kullbackleibler	mutualExclusion	*Hanseniaspora-vineae->Yamadazyma-triangularis*	17.37222
dist_kullbackleibler	mutualExclusion	*Hanseniaspora-nectarophila->Lactobacillus-helveticus*	19.49549
dist_kullbackleibler	mutualExclusion	*Yamadazyma-triangularis->Bacteroides*	17.64776
dist_kullbackleibler	mutualExclusion	*Enterococcus->Penicillium-carneum*	17.41684
correl_spearman	copresence	*Parengyodontium-album->Malassezia-restricta*	0.869356
dist_kullbackleibler	mutualExclusion	*Aureobasidium-pullulans->Issatchenkia-orientalis*	16.97741
dist_kullbackleibler	mutualExclusion	*Lactobacillus-helveticus->Alternaria-metachromatica*	18.44179
dist_kullbackleibler	mutualExclusion	*Kazachstania-unispora->Klebsiella*	16.33148
dist_kullbackleibler	mutualExclusion	*Kazachstania-unispora->Alternaria-metachromatica*	21.04852
dist_kullbackleibler	mutualExclusion	*Naganishia-albida->Lactobacillus-brevis*	17.34381
dist_kullbackleibler	mutualExclusion	*Aspergillus-westerdijkiae->Klebsiella*	16.42153
dist_kullbackleibler	mutualExclusion	*Alternaria-metachromatica->Penicillium-commune*	19.60573
dist_kullbackleibler	mutualExclusion	*Penicillium-carneum->Acinetobacter-uillouiae*	21.24068
dist_bray	mutualExclusion	*Weeksellaceae->Lactobacillus-brevis*	1
dist_kullbackleibler	mutualExclusion	*Penicillium-commune->Klebsiella*	21.90321
dist_kullbackleibler	mutualExclusion	*Kurthiaibsonii->Penicillium-carneum*	24.24137
dist_kullbackleibler	mutualExclusion	*Lactobacillus->Yamadazyma-triangularis*	16.51996
dist_kullbackleibler	mutualExclusion	*Lactobacillus-hamsteri->Alternaria-alternata*	16.73182
correl_pearson	copresence	*Acinetobacter->Acinetobacter-rhizosphaerae*	0.950101
dist_bray	mutualExclusion	*Klebsiella->Pichia-mandshurica*	1
dist_kullbackleibler	mutualExclusion	*Lactobacillus-brevis->Kazachstania-unispora*	16.41972
correl_spearman	mutualExclusion	*Parengyodontium-album->Candida-inconspicua*	-0.62697
dist_kullbackleibler	mutualExclusion	*Cutaneotrichosporon-curvatus->Sporobolomyces-roseus*	17.17647
dist_kullbackleibler	mutualExclusion	*Rhodotorula-mucilaginosa->Enterococcus*	17.04719
dist_kullbackleibler	mutualExclusion	*Aureobasidium-pullulans->Penicillium-commune*	20.48311

dist_kullbackleibler	mutualExclusion	*Issatchenkia-orientalis->Alternaria-alternata*	16.76263
dist_kullbackleibler	mutualExclusion	*Penicillium-carneum->Macrococcus1*	20.47587
dist_kullbackleibler	mutualExclusion	*Kazachstania-unispora->Stemphylium*	20.76204
dist_kullbackleibler	mutualExclusion	*Hanseniaspora-nectarophila->Weeksellaceae*	17.30643
dist_kullbackleibler	mutualExclusion	*Hanseniaspora-vineae->Torulaspora-delbrueckii*	20.91343
correl_spearman	copresence	*Leptobacillium-leptobactrum->Stemphylium*	0.864076
dist_kullbackleibler	mutualExclusion	*Naganishia->Hanseniaspora-nectarophila*	16.36243
dist_kullbackleibler	mutualExclusion	*Stemphylium->Candida-metapsilosis*	16.60956
dist_kullbackleibler	mutualExclusion	*Sporobolomyces-roseus->Lactobacillus-helveticus*	20.48302
dist_bray	mutualExclusion	*Naganishia-albida->Kurthiaibsonii*	1
dist_kullbackleibler	mutualExclusion	*Candida-inconspicua->Penicillium-spinulosum*	16.31331
dist_bray	mutualExclusion	*Kurthiaibsonii->Lactobacillus-hamsteri*	1
dist_kullbackleibler	mutualExclusion	*Penicillium-carneum->Acinetobacter-rhizosphaerae*	22.29976
correl_pearson	copresence	*Acinetobacter2->Klebsiella*	0.951127
dist_kullbackleibler	mutualExclusion	*Torulaspora-delbrueckii->Rhodotorula-mucilaginosa*	17.96895
dist_kullbackleibler	mutualExclusion	*Trichosporon-asahii->Alternaria-metachromatica*	18.68354
dist_kullbackleibler	mutualExclusion	*Lactobacillus-helveticus->Aureobasidium-pullulans*	21.61738
dist_kullbackleibler	mutualExclusion	*Penicillium-carneum->Sporobolomyces-roseus*	24.50777
correl_pearson	copresence	*Acinetobacter-uillouiae->Acinetobacter2*	0.962259
dist_kullbackleibler	mutualExclusion	*Kurthiaibsonii->Lactobacillus-hamsteri*	18.33735
dist_kullbackleibler	mutualExclusion	*Hanseniaspora->Pseudomonas2*	17.45717
dist_kullbackleibler	mutualExclusion	*Alternaria-alternata->Lactobacillus*	17.18306
dist_kullbackleibler	mutualExclusion	*Kurthiaibsonii->Streptococcus-equi*	20.18416
dist_kullbackleibler	mutualExclusion	*Lactobacillus-helveticus->Alternaria-alternata*	19.99912
dist_kullbackleibler	mutualExclusion	*Cutaneotrichosporon-curvatus->Stemphylium*	17.85569
dist_kullbackleibler	mutualExclusion	*Trichosporon-asahii->Botryosphaeria-dothidea*	17.35409

**dist_kullbackleibler**	**mutualExclusion**	***Chromohalobacter->Alternaria-metachromatica***	**18.26729**
**dist_kullbackleibler**	**mutualExclusion**	***Klebsiella->Lactobacillus***	**21.16999**
**dist_kullbackleibler**	**mutualExclusion**	***Yamadazyma-triangularis->Issatchenkia-orientalis***	**17.87574**
**dist_kullbackleibler**	**mutualExclusion**	***Malassezia-globosa->Yamadazyma-triangularis***	**16.60013**
**correl_spearman**	**mutualExclusion**	***Pseudomonas->Aureobasidium-pullulans***	**-0.57586**
**dist_kullbackleibler**	**mutualExclusion**	***Pseudomonas2->Lactobacillus-brevis***	**20.05248**
**dist_kullbackleibler**	**mutualExclusion**	***Naganishia->Botrytis***	**16.50278**
**dist_kullbackleibler**	**mutualExclusion**	***Botryosphaeria-dothidea->Pseudomonas2***	**18.17138**
**dist_kullbackleibler**	**mutualExclusion**	***Naganishia-albida->Macrococcus1***	**17.15395**
**dist_bray**	**mutualExclusion**	***Leuconostoc-mesenteroides->Rikenellaceae***	**1**
**dist_kullbackleibler**	**mutualExclusion**	***Penicillium-spinulosum->Weeksellaceae***	**18.36933**
**dist_kullbackleibler**	**mutualExclusion**	***Botrytis->Cutaneotrichosporon-curvatus***	**21.15508**
**dist_bray**	**mutualExclusion**	***Acidovoraxfacilis->Candida-atlantica***	**1**
**dist_kullbackleibler**	**mutualExclusion**	***Cladosporium-sphaerospermum->Naganishia***	**17.07737**
**dist_kullbackleibler**	**mutualExclusion**	***Aureobasidium-pullulans->Trichosporon-asahii***	**20.14461**
**correl_spearman**	**mutualExclusion**	***Cutaneotrichosporon-curvatus->Hanseniaspora-nectarophila***	**-0.62377**
**correl_spearman**	**mutualExclusion**	***Cutaneotrichosporon-curvatus->Erysiphe-necator***	**-0.68331**
**dist_kullbackleibler**	**mutualExclusion**	***Penicillium-spinulosum->Lactobacillus-hamsteri***	**16.66943**
**dist_kullbackleibler**	**mutualExclusion**	***Lactobacillus-hamsteri->Botrytis***	**18.82824**
**dist_kullbackleibler**	**mutualExclusion**	***Parengyodontium-album->Pseudomonas***	**16.93989**
**dist_kullbackleibler**	**mutualExclusion**	***Trichosporon-asahii->Acinetobacter-johnsonii***	**16.95515**
**correl_spearman**	**copresence**	***Leptobacillium-leptobactrum->Parengyodontium-album***	**0.919422**
**dist_kullbackleibler**	**mutualExclusion**	***Candida-metapsilosis->Aureobasidium-pullulans***	**17.61388**
**dist_kullbackleibler**	**mutualExclusion**	***Enhydrobacter->Acinetobacter-rhizosphaerae***	**16.64243**
**dist_kullbackleibler**	**mutualExclusion**	***Kazachstania-unispora->Sporobolomyces-roseus***	**16.96012**
**dist_bray**	**mutualExclusion**	***Weeksellaceae->Enhydrobacter-***	**1**
**dist_kullbackleibler**	**mutualExclusion**	***Rhodotorula-mucilaginosa->Candida-atlantica***	**16.80767**

**dist_kullbackleibler**	**mutualExclusion**	***Lactobacillus-brevis->Meyerozyma-guilliermondii***	**18.0477**
**correl_spearman**	**copresence**	***Hanseniaspora->Lachancea-thermotolerans***	**0.831086**
**dist_kullbackleibler**	**mutualExclusion**	***Weeksellaceae->Yamadazyma-triangularis***	**19.75982**
**dist_bray**	**mutualExclusion**	***Acinetobacter-uillouiae->Pichia-mandshurica***	**1**
**dist_kullbackleibler**	**mutualExclusion**	***Sporobolomyces-roseus->Leuconostoc1***	**16.9834**
**dist_kullbackleibler**	**mutualExclusion**	***Lactobacillus->Erysiphe-necator***	**17.03031**
**dist_kullbackleibler**	**mutualExclusion**	***Torulaspora-delbrueckii->Corynebacterium-variabile***	**16.23789**
**dist_kullbackleibler**	**mutualExclusion**	***Klebsiella->Candida-diddensiae***	**16.85253**
**dist_kullbackleibler**	**mutualExclusion**	***Alternaria-metachromatica->Lactobacillus***	**18.1663**
**dist_kullbackleibler**	**mutualExclusion**	***Lactobacillus-zeae->Alternaria-alternata***	**16.24824**
**dist_kullbackleibler**	**mutualExclusion**	***Kurthiaibsonii->Enhydrobacter***	**19.06513**
**dist_kullbackleibler**	**mutualExclusion**	***Cladosporium-tenuissimum->Yamadazyma-triangularis***	**16.29436**
**dist_kullbackleibler**	**mutualExclusion**	***Meyerozyma-guilliermondii->Torulaspora-delbrueckii***	**20.36701**
**dist_kullbackleibler**	**mutualExclusion**	***Botrytis->Candida-metapsilosis***	**16.4991**
**dist_kullbackleibler**	**mutualExclusion**	***Parengyodontium-album->Pediococcus***	**17.41118**
**dist_kullbackleibler**	**mutualExclusion**	***Yamadazyma-triangularis->Mycosphaerella-tassiana***	**19.30948**
**dist_bray**	**mutualExclusion**	***Candida-atlantica->Pseudomonas2***	**1**
**dist_kullbackleibler**	**mutualExclusion**	***Acinetobacter-uillouiae->Pichia-mandshurica***	**16.98817**
**dist_kullbackleibler**	**mutualExclusion**	***Chromohalobacter->Enhydrobacter-***	**17.32909**
**dist_kullbackleibler**	**mutualExclusion**	***Enterococcus->Penicillium-commune***	**18.07368**
**dist_kullbackleibler**	**mutualExclusion**	***Issatchenkia-orientalis->Kurthiaibsonii***	**16.53992**
**dist_kullbackleibler**	**mutualExclusion**	***Hanseniaspora->Chromohalobacter***	**19.09328**
**correl_spearman**	**mutualExclusion**	***Lactobacillus-helveticus->Parengyodontium-album***	**-0.57169**
**dist_kullbackleibler**	**mutualExclusion**	***Kurthiaibsonii->Pseudomonas2***	**18.40785**
**dist_kullbackleibler**	**mutualExclusion**	***Leuconostoc-mesenteroides->Naganishia***	**17.22719**
**dist_kullbackleibler**	**mutualExclusion**	***Naganishia->Parengyodontium-album***	**17.50342**
**dist_kullbackleibler**	**mutualExclusion**	***Lactobacillus-brevis->Trichosporon-asahii***	**17.06678**

dist_kullbackleibler	mutualExclusion	*Kurthiaibsonii->Weeksellaceae*	20.54852
dist_kullbackleibler	mutualExclusion	*Penicillium-carneum->Erysiphe-necator*	25.05318
dist_kullbackleibler	mutualExclusion	*Chromohalobacter->Acinetobacter-johnsonii*	17.85701
dist_kullbackleibler	mutualExclusion	*Kazachstania-unispora->Aureobasidium-pullulans*	22.80958
dist_kullbackleibler	mutualExclusion	*Rhodotorula-mucilaginosa->Lactobacillus-brevis*	19.34944
dist_kullbackleibler	mutualExclusion	*Sporobolomyces-roseus->Naganishia-albida*	17.87757
correl_pearson	mutualExclusion	*Lactobacillus-helveticus->Hanseniaspora*	-0.43802
dist_kullbackleibler	mutualExclusion	*Naganishia-albida->Torulaspora-delbrueckii*	21.56926
dist_kullbackleibler	mutualExclusion	*Penicillium->Yamadazyma-triangularis*	18.29966
dist_bray	mutualExclusion	*Enhydrobacter-->Pediococcus*	1
dist_kullbackleibler	mutualExclusion	*Issatchenkia-orientalis->Erysiphe-necator*	16.44995
dist_kullbackleibler	mutualExclusion	*Stemphylium->Lactobacillus-helveticus*	19.50141
dist_kullbackleibler	mutualExclusion	*Saccharomyces->Torulaspora-delbrueckii*	17.38607
dist_kullbackleibler	mutualExclusion	*Naganishia->Enterococcus*	17.83186
dist_kullbackleibler	mutualExclusion	*Enterococcus->Penicillium*	16.80875
dist_kullbackleibler	mutualExclusion	*Candida-inconspicua->Torulaspora-delbrueckii*	18.60973
dist_kullbackleibler	mutualExclusion	*Alternaria-metachromatica->Candida-metapsilosis*	16.35299
dist_bray	copresence	*Acinetobacter2->Klebsiella*	0.253052
dist_kullbackleibler	mutualExclusion	*Alternaria-alternata->Kazachstania-unispora*	22.32371
dist_kullbackleibler	mutualExclusion	*Pseudomonas2->Aureobasidium-pullulans*	20.02388
dist_kullbackleibler	mutualExclusion	*Acinetobacter-rhizosphaerae->Penicillium-commune*	22.71418
dist_kullbackleibler	mutualExclusion	*Wickerhamomyces-anomalus->Enterococcus*	16.14942
dist_kullbackleibler	mutualExclusion	*Kluyveromyces-marxianus->Lactobacillus-brevis*	19.33327
dist_kullbackleibler	mutualExclusion	*Lactobacillus-helveticus->Enhydrobacter-*	18.55
dist_kullbackleibler	mutualExclusion	*Alternaria-alternata->Cutaneotrichosporon-curvatus*	19.56513
dist_kullbackleibler	mutualExclusion	*Penicillium-spinulosum->Pseudomonas*	16.69566

dist_kullbackleibler	mutualExclusion	*Kurthiaibsonii->Kazachstania-unispora*	22.58212
dist_kullbackleibler	mutualExclusion	*Lactobacillus-helveticus->Erysiphe-necator*	21.99604
dist_kullbackleibler	mutualExclusion	*Lactobacillus->Botrytis*	18.21968
dist_kullbackleibler	mutualExclusion	*Penicillium-spinulosum->Cutaneotrichosporon-curvatus*	19.02922
correl_pearson	mutualExclusion	*Cutaneotrichosporon-curvatus->Hanseniaspora-nectarophila*	-0.34374
dist_kullbackleibler	mutualExclusion	*Parengyodontium-album->Issatchenkia-orientalis*	17.92639
dist_kullbackleibler	mutualExclusion	*Hanseniaspora-vineae->Enterococcus*	18.24196
dist_kullbackleibler	mutualExclusion	*Lactobacillus-brevis->Candida-inconspicua*	20.23329
dist_kullbackleibler	mutualExclusion	*Weeksellaceae->Lactobacillus-brevis*	20.09547
dist_kullbackleibler	mutualExclusion	*Candida-metapsilosis->Erysiphe-necator*	16.5113
dist_kullbackleibler	mutualExclusion	*Hanseniaspora-nectarophila->Kazachstania-unispora*	22.60503
dist_kullbackleibler	mutualExclusion	*Parengyodontium-album->Kazachstania-unispora*	22.21105
dist_kullbackleibler	mutualExclusion	*Hanseniaspora->Kazachstania-unispora*	21.8705
dist_kullbackleibler	mutualExclusion	*Hanseniaspora->Candida-metapsilosis*	17.92787
dist_kullbackleibler	mutualExclusion	*Acinetobacter-rhizosphaerae->Pseudomonas2*	18.43292
dist_kullbackleibler	mutualExclusion	*Trichosporon-asahii->Parengyodontium-album*	23.06997
dist_kullbackleibler	mutualExclusion	*Penicillium->Torulaspora-delbrueckii*	19.03463
dist_kullbackleibler	mutualExclusion	*Kurthiaibsonii->Pediococcus*	20.68977
dist_kullbackleibler	mutualExclusion	*Chromohalobacter->Klebsiella*	17.25966
dist_kullbackleibler	mutualExclusion	*Penicillium-spinulosum->Kazachstania-unispora*	21.89649
dist_kullbackleibler	mutualExclusion	*Pediococcus->Torulaspora-delbrueckii*	19.47255
dist_kullbackleibler	mutualExclusion	*Penicillium-spinulosum->Candida-metapsilosis*	16.99771
dist_kullbackleibler	mutualExclusion	*Lactobacillus-helveticus->Botrytis*	21.76329
dist_kullbackleibler	mutualExclusion	*Torulaspora-delbrueckii->Filobasidium-unidentified*	16.2147
dist_kullbackleibler	mutualExclusion	*Saccharomyces->Cladosporium-sphaerospermum*	16.47572
dist_kullbackleibler	mutualExclusion	*Acidovoraxfacilis->Candida-atlantica*	17.46607

**dist_kullbackleibler**	**mutualExclusion**	***Trichosporon-asahii->Penicillium-spinulosum***	**17.99898**
**dist_kullbackleibler**	**mutualExclusion**	***Naganishia-albida->Erysiphe-necator***	**16.85026**
**dist_kullbackleibler**	**mutualExclusion**	***Pseudomonas->Lactobacillus-brevis***	**19.2657**
**dist_kullbackleibler**	**mutualExclusion**	***Penicillium-spinulosum->Pediococcus***	**16.85059**
**dist_kullbackleibler**	**mutualExclusion**	***Kazachstania-unispora->Cladosporium-sphaerospermum***	**20.74987**
**dist_kullbackleibler**	**mutualExclusion**	***Aspergillus-westerdijkiae->Yamadazyma-triangularis***	**20.35256**
**dist_kullbackleibler**	**mutualExclusion**	***Yamadazyma-triangularis->Acidovoraxfacilis***	**16.15604**
**dist_kullbackleibler**	**mutualExclusion**	***Leuconostoc-mesenteroides->Rhodotorula-mucilaginosa***	**16.79335**
**correl_spearman**	**mutualExclusion**	***Cutaneotrichosporon-curvatus->Stemphylium***	**-0.63817**
**dist_kullbackleibler**	**mutualExclusion**	***Lactobacillus-hamsteri->Erysiphe-necator***	**16.91026**
**dist_kullbackleibler**	**mutualExclusion**	***Filobasidium-unidentified->Leuconostoc-mesenteroides***	**17.31941**
**correl_spearman**	**mutualExclusion**	***Lactobacillus-helveticus->Hanseniaspora-nectarophila***	**-0.6538**
**dist_kullbackleibler**	**mutualExclusion**	***Chromohalobacter->Stemphylium***	**18.25228**
**dist_kullbackleibler**	**mutualExclusion**	***Torulaspora-delbrueckii->Carnobacterium***	**22.70465**
**correl_spearman**	**mutualExclusion**	***Lactobacillus-helveticus->Erysiphe-necator***	**-0.59086**
**dist_kullbackleibler**	**mutualExclusion**	***Erysiphe-necator->Lactobacillus-zeae***	**17.02478**
**dist_kullbackleibler**	**mutualExclusion**	***Pseudomonas2->Sporobolomyces-roseus***	**19.77969**
**correl_spearman**	**mutualExclusion**	***Hanseniaspora->Lactobacillus-helveticus***	**-0.63655**
**dist_kullbackleibler**	**mutualExclusion**	***Acinetobacter-uillouiae->Penicillium-commune***	**18.8288**
**dist_kullbackleibler**	**mutualExclusion**	***Enterococcus->Carnobacterium***	**17.8369**
**correl_pearson**	**mutualExclusion**	***Lactobacillus-helveticus->Stemphylium***	**-0.38342**
**correl_pearson**	**copresence**	***Acinetobacter->Acinetobacter-uillouiae***	**0.951107**
**dist_kullbackleibler**	**mutualExclusion**	***Penicillium-carneum->Yamadazyma-triangularis***	**21.10002**
**dist_kullbackleibler**	**mutualExclusion**	***Yamadazyma-triangularis->Naganishia-albida***	**18.70455**
**dist_kullbackleibler**	**mutualExclusion**	***Trichosporon-asahii->Stemphylium***	**19.38262**
**dist_kullbackleibler**	**mutualExclusion**	***Candida-atlantica->Kazachstania-unispora***	**18.13089**
**dist_kullbackleibler**	**mutualExclusion**	***Botrytis->Issatchenkia-orientalis***	**16.58989**

dist_kullbackleibler	mutualExclusion	*Streptococcus-equi->Acinetobacter-rhizosphaerae*	18.8565
dist_kullbackleibler	mutualExclusion	*Pediococcus->Sporobolomyces-roseus*	16.85306
dist_kullbackleibler	mutualExclusion	*Issatchenkia-orientalis->Hanseniaspora-nectarophila*	17.91812
dist_kullbackleibler	mutualExclusion	*Acinetobacter-uillouiae->Chromohalobacter*	17.06159
dist_kullbackleibler	mutualExclusion	*Lactobacillus-helveticus->Hanseniaspora*	19.54887
dist_kullbackleibler	mutualExclusion	*Penicillium-spinulosum->Chromohalobacter*	19.97156
dist_bray	mutualExclusion	*Macrococcus1->Lactobacillus-hamsteri*	1
dist_kullbackleibler	mutualExclusion	*Naganishia-albida->Kurthiaibsonii*	21.61111
dist_kullbackleibler	mutualExclusion	*Acinetobacter-rhizosphaerae->Lactobacillus-helveticus*	21.0936
dist_kullbackleibler	mutualExclusion	*Botryosphaeria-dothidea->Naganishia-albida*	16.16689
dist_kullbackleibler	mutualExclusion	*Acinetobacter-johnsonii->Lactobacillus*	17.30007
dist_kullbackleibler	mutualExclusion	*Acinetobacter-johnsonii->Penicillium-carneum*	20.36279
dist_kullbackleibler	mutualExclusion	*Penicillium-commune->Sporobolomyces-roseus*	20.68392
dist_kullbackleibler	mutualExclusion	*Penicillium-carneum->Botryosphaeria-dothidea*	22.13064
dist_kullbackleibler	mutualExclusion	*Erysiphe-necator->Penicillium-commune*	21.77231
dist_kullbackleibler	mutualExclusion	*Kurthiaibsonii->Bacteroides*	16.63764
dist_kullbackleibler	mutualExclusion	*Parengyodontium-album->Kluyveromyces-marxianus*	18.01668
dist_kullbackleibler	mutualExclusion	*Aureobasidium-pullulans->Naganishia-albida*	17.48915
dist_kullbackleibler	mutualExclusion	*Parengyodontium-album->Naganishia-albida*	18.27476
dist_kullbackleibler	mutualExclusion	*Penicillium-carneum->Torulaspora-delbrueckii*	19.98511
dist_kullbackleibler	mutualExclusion	*Chromohalobacter->Erysiphe-necator*	20.68909
dist_kullbackleibler	mutualExclusion	*Lactobacillus-delbrueckii->Klebsiella*	16.61223
dist_bray	copresence	*Acinetobacter2->Acinetobacter-johnsonii*	0.245093
dist_kullbackleibler	mutualExclusion	*Staphylococcus-equorum->Leuconostoc-mesenteroides*	18.19106
dist_kullbackleibler	mutualExclusion	*Carnobacterium->Lactobacillus-brevis*	20.15453
dist_kullbackleibler	mutualExclusion	*Leuconostoc-mesenteroides->Saccharomyces*	17.58842

**dist_kullbackleibler**	**mutualExclusion**	***Kurthiaibsonii->Lactobacillus***	**19.6240 3**
**dist_kullbackleibler**	**mutualExclusion**	***Lactobacillus->Acinetobacter-rhizosphaerae***	**18.7643 1**
**dist_kullbackleibler**	**mutualExclusion**	***Acinetobacter-johnsonii->Kazachstania-unispora***	**16.2946**
**dist_bray**	**mutualExclusion**	***Lactobacillus-hamsteri->Hanseniaspora***	**1**
**dist_kullbackleibler**	**mutualExclusion**	***Yamadazyma-triangularis->Candida-diddensiae***	**18.7589 4**
**dist_kullbackleibler**	**mutualExclusion**	***Candida-atlantica->Issatchenkia-orientalis***	**16.3626 8**
**dist_kullbackleibler**	**mutualExclusion**	***Staphylococcus-equorum->Lactobacillus-brevis***	**19.8398**
**dist_kullbackleibler**	**mutualExclusion**	***Carnobacterium->Penicillium-spinulosum***	**16.9336 6**
**dist_kullbackleibler**	**mutualExclusion**	***Pseudomonas2->Macrococcus1***	**17.1180 6**
**dist_kullbackleibler**	**mutualExclusion**	***Lactobacillus->Acinetobacter-uillouiae***	**17.3289 8**
**dist_kullbackleibler**	**mutualExclusion**	***Penicillium-carneum->Alternaria-metachromatica***	**21.4674 8**
**dist_kullbackleibler**	**mutualExclusion**	***Pseudomonas2->Erysiphe-necator***	**20.4513 5**
**correl_spearman**	**mutualExclusion**	***Lactobacillus-helveticus->Alternaria-alternata***	**-0.62384**
**dist_kullbackleibler**	**mutualExclusion**	***Carnobacterium->Enhydrobacter-***	**18.8821 5**
**correl_spearman**	**copresence**	***Botryosphaeria-dothidea->Leptobacillium-leptobactrum***	**0.82602 1**
**dist_bray**	**mutualExclusion**	***Candida-atlantica->Lactobacillus-hamsteri***	**1**
**dist_kullbackleibler**	**mutualExclusion**	***Weeksellaceae->Macrococcus1***	**16.4478 1**
**dist_kullbackleibler**	**mutualExclusion**	***Chromohalobacter->Kurthiaibsonii***	**16.7227 6**
**dist_kullbackleibler**	**mutualExclusion**	***Botrytis->Penicillium-commune***	**21.9949 5**
**dist_kullbackleibler**	**mutualExclusion**	***Naganishia->Yamadazyma-triangularis***	**19.7592 3**
**dist_bray**	**mutualExclusion**	***Lactobacillus-brevis->Pseudomonas2***	**1**
**dist_kullbackleibler**	**mutualExclusion**	***Naganishia->Candida-atlantica***	**18.2284 4**
**dist_kullbackleibler**	**mutualExclusion**	***Candida-atlantica->Aspergillus-westerdijkiae***	**17.1236 6**
**dist_kullbackleibler**	**mutualExclusion**	***Trichosporon-asahii->Candida-atlantica***	**17.6183 2**
**dist_kullbackleibler**	**mutualExclusion**	***Trichosporon-asahii->Yamadazyma-triangularis***	**16.5371 8**
**dist_kullbackleibler**	**mutualExclusion**	***Acinetobacter-uillouiae->Kazachstania-unispora***	**16.2504 6**

dist_kullbackleibler	mutualExclusion	*Alternaria-alternata->Naganishia-albida*	19.02619
dist_kullbackleibler	mutualExclusion	*Acinetobacter-rhizosphaerae->Cutaneotrichosporon-curvatus*	19.79259
dist_kullbackleibler	mutualExclusion	*Pseudomonas->Hanseniaspora-nectarophila*	16.48173
dist_kullbackleibler	mutualExclusion	*Chromohalobacter->Botrytis*	19.66169
correl_spearman	copresence	*Stemphylium->Lachancea-thermotolerans*	0.834439
dist_kullbackleibler	mutualExclusion	*Acinetobacter-uillouiae->Cutaneotrichosporon-curvatus*	17.07929
dist_kullbackleibler	mutualExclusion	*Lactobacillus-brevis->Kocuria*	18.31735
dist_bray	mutualExclusion	*Botryosphaeria-dothidea->Pseudomonas2*	1
dist_kullbackleibler	mutualExclusion	*Filobasidium-unidentified->Lactobacillus-brevis*	18.01102
dist_kullbackleibler	mutualExclusion	*Issatchenkia-orientalis->Alternaria-metachromatica*	16.26796
dist_kullbackleibler	mutualExclusion	*Yamadazyma-triangularis->Pichia-mandshurica*	16.68317
dist_kullbackleibler	mutualExclusion	*Hanseniaspora->Trichosporon-asahii*	18.71013
dist_kullbackleibler	mutualExclusion	*Pseudomonas2->Penicillium-spinulosum*	19.5346
dist_kullbackleibler	mutualExclusion	*Yamadazyma-triangularis->Candida-magnoliae*	16.95778
dist_kullbackleibler	mutualExclusion	*Pseudomonas2->Botrytis*	19.69947
dist_kullbackleibler	mutualExclusion	*Botryosphaeria-dothidea->Carnobacterium*	16.1436
dist_kullbackleibler	mutualExclusion	*Candida-inconspicua->Erysiphe-necator*	16.17473
dist_bray	mutualExclusion	*Aspergillus-westerdijkiae->Torulaspora-delbrueckii*	1
dist_kullbackleibler	mutualExclusion	*Candida-atlantica->Penicillium-carneum*	19.4472
dist_kullbackleibler	mutualExclusion	*Candida-atlantica->Cutaneotrichosporon-curvatus*	16.19069
dist_kullbackleibler	mutualExclusion	*Cutaneotrichosporon-curvatus->Aureobasidium-pullulans*	20.7767
dist_bray	mutualExclusion	*Candida-atlantica->Naganishia*	1
dist_kullbackleibler	mutualExclusion	*Yamadazyma-triangularis->Penicillium-commune*	24.17528
dist_kullbackleibler	mutualExclusion	*Hanseniaspora->Issatchenkia-orientalis*	16.40403
dist_kullbackleibler	mutualExclusion	*Penicillium-carneum->Lactobacillus-brevis*	19.76222
dist_kullbackleibler	mutualExclusion	*Candida-atlantica->Lactobacillus-delbrueckii*	16.62197
dist_kullbackleibler	mutualExclusion	*Parengyodontium-album->Lactobacillus-zeae*	19.34095

dist_kullbackleibler	mutualExclusion	*Hanseniaspora-nectarophila->Lactobacillus-hamsteri*	17.20778
dist_kullbackleibler	mutualExclusion	*Macrococcus1->Lactobacillus-delbrueckii*	16.74045
dist_kullbackleibler	mutualExclusion	*Pseudomonas2->Candida-atlantica*	18.99861
dist_kullbackleibler	mutualExclusion	*Weeksellaceae->Botrytis*	16.67611
dist_bray	mutualExclusion	*Enhydrobacter-->Penicillium-carneum*	1
correl_pearson	mutualExclusion	*Lactobacillus-helveticus->Hanseniaspora-nectarophila*	-0.44295
correl_pearson	mutualExclusion	*Lactobacillus-helveticus->Botryosphaeria-dothidea*	-0.37964
dist_kullbackleibler	mutualExclusion	*Erysiphe-necator->Trichosporon-asahii*	20.2696
dist_kullbackleibler	mutualExclusion	*Leuconostoc-mesenteroides->Rikenellaceae*	17.06584
dist_kullbackleibler	mutualExclusion	*Candida-metapsilosis->Hanseniaspora-nectarophila*	17.08417
dist_bray	mutualExclusion	*Kazachstania-unispora->Kurthiaibsonii*	1
dist_kullbackleibler	mutualExclusion	*Trichosporon-asahii->Sporobolomyces-roseus*	17.664
dist_bray	mutualExclusion	*Naganishia-albida->Enhydrobacter-*	1
dist_kullbackleibler	mutualExclusion	*Chromohalobacter->Sporobolomyces-roseus*	18.95404
dist_kullbackleibler	mutualExclusion	*Naganishia-albida->Hanseniaspora-nectarophila*	17.25619
dist_kullbackleibler	mutualExclusion	*Klebsiella->Penicillium-carneum*	21.29127
correl_spearman	mutualExclusion	*Lactobacillus-helveticus->Aureobasidium-pullulans*	-0.66983
dist_kullbackleibler	mutualExclusion	*Naganishia-albida->Klebsiella*	19.93681
dist_kullbackleibler	mutualExclusion	*Hanseniaspora->Candida-inconspicua*	16.21116
dist_kullbackleibler	mutualExclusion	*Lactobacillus-helveticus->Kurthiaibsonii*	22.84379
dist_bray	mutualExclusion	*Weeksellaceae->Kurthiaibsonii*	1
dist_kullbackleibler	mutualExclusion	*Lactobacillus-helveticus->Acinetobacter-uillouiae*	18.44182
dist_kullbackleibler	mutualExclusion	*Pseudomonas2->Parengyodontium-album*	20.93366
dist_kullbackleibler	mutualExclusion	*Acinetobacter-johnsonii->Pseudomonas2*	16.21178
dist_kullbackleibler	mutualExclusion	*Pseudomonas->Torulaspora-delbrueckii*	21.01449
correl_spearman	mutualExclusion	*Penicillium-spinulosum->Lactobacillus-helveticus*	-0.69584
dist_kullbackleibler	mutualExclusion	*Torulaspora-delbrueckii->Staphylococcus-equorum*	22.03761

dist_kullbackleibler	mutualExclusion	*Erysiphe-necator->Weeksellaceae*	17.28343
dist_kullbackleibler	mutualExclusion	*Issatchenkia-orientalis->Torulaspora-delbrueckii*	19.87939
dist_kullbackleibler	mutualExclusion	*Staphylococcus2->Lactobacillus-brevis*	16.14179
dist_kullbackleibler	mutualExclusion	*Macrococcus1->Penicillium-commune*	18.85692
dist_kullbackleibler	mutualExclusion	*Acinetobacter-rhizosphaerae->Naganishia-albida*	20.38953
dist_kullbackleibler	mutualExclusion	*Alternaria-metachromatica->Pseudomonas2*	18.27136
dist_kullbackleibler	mutualExclusion	*Hanseniaspora-nectarophila->Chromohalobacter*	19.76883
dist_kullbackleibler	mutualExclusion	*Weeksellaceae->Enhydrobacter-*	19.04324
dist_kullbackleibler	mutualExclusion	*Macrococcus1->Naganishia*	17.52043
correl_spearman	mutualExclusion	*Pseudomonas->Penicillium-spinulosum*	-0.57465
dist_kullbackleibler	mutualExclusion	*Rhodotorula-mucilaginosa->Yamadazyma-triangularis*	21.9156
dist_kullbackleibler	mutualExclusion	*Naganishia-albida->Botrytis*	17.95702
dist_kullbackleibler	mutualExclusion	*Acinetobacter-rhizosphaerae->Weeksellaceae*	19.77875
dist_kullbackleibler	mutualExclusion	*Botryosphaeria-dothidea->Chromohalobacter*	16.31483
dist_kullbackleibler	mutualExclusion	*Lactobacillus-brevis->Penicillium-commune*	17.05399
dist_kullbackleibler	mutualExclusion	*Chromohalobacter->Cladosporium-sphaerospermum*	17.89142
dist_kullbackleibler	mutualExclusion	*Aureobasidium-pullulans->Chromohalobacter*	20.63033
dist_bray	mutualExclusion	*Enhydrobacter-->Lactobacillus-hamsteri*	1
dist_kullbackleibler	mutualExclusion	*Klebsiella->Rikenellaceae*	17.3875
dist_kullbackleibler	mutualExclusion	*Yamadazyma-triangularis->Leptobacillium-leptobactrum*	17.93799
dist_kullbackleibler	mutualExclusion	*Alternaria-alternata->Pseudomonas2*	20.02885
dist_kullbackleibler	mutualExclusion	*Torulaspora-delbrueckii->Pseudomonas2*	21.35951
dist_kullbackleibler	mutualExclusion	*Candida-metapsilosis->Lactobacillus-brevis*	19.6081
dist_bray	copresence	*Hanseniaspora->Candida-stellata*	0.238821
dist_kullbackleibler	mutualExclusion	*Weeksellaceae->Aureobasidium-pullulans*	16.83019
dist_kullbackleibler	mutualExclusion	*Botrytis->Lactobacillus-zeae*	16.87827
dist_bray	copresence	*Cladosporium-tenuissimum->Aureobasidium-pullulans*	0.233273

**correl_spearman**	**mutualExclusion**	***Lactobacillus-helveticus->Stemphylium***	**-0.6459**
**dist_kullbackleibler**	**mutualExclusion**	***Candida-diddensiae->Kurthiaibsonii***	**16.72074**
**dist_kullbackleibler**	**mutualExclusion**	***Lactobacillus-brevis->Lactobacillus-helveticus***	**16.21009**
**dist_kullbackleibler**	**mutualExclusion**	***Saccharomyces->Yamadazyma-triangularis***	**16.34386**
**dist_kullbackleibler**	**mutualExclusion**	***Lactobacillus-zeae->Aureobasidium-pullulans***	**16.52855**
**dist_kullbackleibler**	**mutualExclusion**	***Penicillium-commune->Candida-atlantica***	**16.83228**
**dist_kullbackleibler**	**mutualExclusion**	***Parengyodontium-album->Candida-inconspicua***	**19.38779**
**dist_bray**	**mutualExclusion**	***Botrytis->Lactobacillus-hamsteri***	**1**
**dist_kullbackleibler**	**mutualExclusion**	***Pichia-mandshurica->Klebsiella***	**18.67183**
**dist_kullbackleibler**	**mutualExclusion**	***Trichosporon-asahii->Enterococcus***	**16.52558**
**correl_spearman**	**copresence**	***Malassezia-restricta->Alternaria-alternata***	**0.830257**
**dist_kullbackleibler**	**mutualExclusion**	***Leuconostoc-mesenteroides->Penicillium-carneum***	**19.5102**
**dist_kullbackleibler**	**mutualExclusion**	***Acinetobacter-uillouiae->Naganishia-albida***	**19.04201**
**dist_kullbackleibler**	**mutualExclusion**	***Penicillium-carneum->Hanseniaspora***	**23.221**
**dist_kullbackleibler**	**mutualExclusion**	***Kazachstania-unispora->Erysiphe-necator***	**22.92426**
**dist_kullbackleibler**	**mutualExclusion**	***Torulaspora-delbrueckii->Lachancea-thermotolerans***	**19.72829**
**correl_spearman**	**copresence**	***Hanseniaspora->Aspergillus***	**0.832366**
**dist_kullbackleibler**	**mutualExclusion**	***Candida-metapsilosis->Alternaria-alternata***	**16.85347**
**dist_kullbackleibler**	**mutualExclusion**	***Hanseniaspora->Naganishia***	**18.7431**
**dist_kullbackleibler**	**mutualExclusion**	***Enterococcus->Staphylococcus-equorum***	**18.72372**
**correl_pearson**	**copresence**	***Acinetobacter-johnsonii->Acinetobacter2***	**0.958043**
**dist_kullbackleibler**	**mutualExclusion**	***Naganishia-albida->Alternaria-metachromatica***	**19.21722**
**dist_kullbackleibler**	**mutualExclusion**	***Yamadazyma-triangularis->Lactobacillus-hamsteri***	**17.81429**
**dist_kullbackleibler**	**mutualExclusion**	***Candida-metapsilosis->Parengyodontium-album***	**20.21123**
**dist_kullbackleibler**	**mutualExclusion**	***Macrococcus1->Trichosporon-asahii***	**18.3054**
**dist_bray**	**mutualExclusion**	***Weeksellaceae->Sporobolomyces-roseus***	**1**
**dist_bray**	**mutualExclusion**	***Weeksellaceae->Acinetobacter-rhizosphaerae***	**1**
**dist_kullbackleibler**	**mutualExclusion**	***Candida-metapsilosis->Cladosporium-sphaerospermum***	**17.71429**

dist_kullbackleibler	mutualExclusion	*Lactobacillus-helveticus->Penicillium-spinulosum*	22.4218
dist_kullbackleibler	mutualExclusion	*Kurthiaibsonii->Cutaneotrichosporon-curvatus*	21.4566
dist_kullbackleibler	mutualExclusion	*Filobasidium-unidentified->Yamadazyma-triangularis*	18.62163
dist_kullbackleibler	mutualExclusion	*Acinetobacter-rhizosphaerae->Kazachstania-unispora*	21.51925
dist_kullbackleibler	mutualExclusion	*Cladosporium-sphaerospermum->Lactobacillus*	17.831
dist_kullbackleibler	mutualExclusion	*Cutaneotrichosporon-curvatus->Enhydrobacter-*	18.00867
dist_kullbackleibler	mutualExclusion	*Trichosporon-asahii->Botrytis*	19.37599
dist_kullbackleibler	mutualExclusion	*Penicillium-spinulosum->Issatchenkia-orientalis*	16.45648
dist_kullbackleibler	mutualExclusion	*Acinetobacter-uillouiae->Pediococcus*	18.10835
dist_kullbackleibler	mutualExclusion	*Lactobacillus->Candida-atlantica*	19.20752
dist_kullbackleibler	mutualExclusion	*Penicillium-commune->Acinetobacter-johnsonii*	21.4647
dist_kullbackleibler	mutualExclusion	*Lactobacillus-brevis->Acidovoraxfacilis*	16.24044
dist_kullbackleibler	mutualExclusion	*Torulaspora-delbrueckii->Aspergillus-westerdijkiae*	22.44189
dist_kullbackleibler	mutualExclusion	*Pseudomonas2->Yamadazyma-triangularis*	17.26601
dist_kullbackleibler	mutualExclusion	*Wickerhamomyces-anomalus->Yamadazyma-triangularis*	20.07611
dist_kullbackleibler	mutualExclusion	*Kazachstania-unispora->Botryosphaeria-dothidea*	17.89459
dist_kullbackleibler	mutualExclusion	*Enterococcus->Aspergillus-westerdijkiae*	19.29125
dist_kullbackleibler	mutualExclusion	*Kurthiaibsonii->Carnobacterium*	16.14984
dist_kullbackleibler	mutualExclusion	*Weeksellaceae->Stemphylium*	16.27096
dist_kullbackleibler	mutualExclusion	*Penicillium-carneum->Parengyodontium-album*	21.38465
dist_kullbackleibler	mutualExclusion	*Penicillium-commune->Alternaria-alternata*	20.73491
dist_kullbackleibler	mutualExclusion	*Cutaneotrichosporon-curvatus->Erysiphe-necator*	20.90038
dist_kullbackleibler	mutualExclusion	*Enhydrobacter-->Lactobacillus-hamsteri*	16.83252
dist_kullbackleibler	mutualExclusion	*Penicillium-carneum->Alternaria-alternata*	23.14314
dist_kullbackleibler	mutualExclusion	*Enhydrobacter-->Naganishia-albida*	20.10575

**dist_kullbackleibler**	**mutualExclusion**	***Botrytis->Penicillium-carneum***	**23.70631**
**dist_kullbackleibler**	**mutualExclusion**	***Hanseniaspora->Pediococcus***	**16.60273**
**dist_kullbackleibler**	**mutualExclusion**	***Alternaria-metachromatica->Cutaneotrichosporon-curvatus***	**19.87247**
**dist_bray**	**mutualExclusion**	***Pseudomonas2->Sporobolomyces-roseus***	**1**
**dist_kullbackleibler**	**mutualExclusion**	***Alternaria-alternata->Weeksellaceae***	**17.06551**
**correl_spearman**	**mutualExclusion**	***Cutaneotrichosporon-curvatus->Parengyodontium-album***	**-0.61776**
**dist_kullbackleibler**	**mutualExclusion**	***Mannheimia->Yamadazyma-triangularis***	**16.5802**
**dist_kullbackleibler**	**mutualExclusion**	***Macrococcus->Yamadazyma-triangularis***	**16.90154**
**dist_kullbackleibler**	**mutualExclusion**	***Yamadazyma-triangularis->Leuconostoc***	**16.64826**
**dist_kullbackleibler**	**mutualExclusion**	***Botrytis->Kazachstania-unispora***	**22.26068**
**dist_kullbackleibler**	**mutualExclusion**	***Hanseniaspora-nectarophila->Trichosporon-asahii***	**18.14988**
**dist_kullbackleibler**	**mutualExclusion**	***Parengyodontium-album->Cutaneotrichosporon-curvatus***	**19.86003**
**correl_spearman**	**mutualExclusion**	***Lactobacillus-helveticus->Botrytis***	**-0.62894**

Table S7. Identified interactions between microbes in Cypriot cheeses in the co-occurrence network.

Cooccurance_method	Interaction Type	Interacting Microbes	weight
**correl_spearman**	**mutualExclusion**	***Hanseniaspora-nectarophila->Lactococcus***	**-0.70454545**
**dist_kullbackleibler**	**mutualExclusion**	***Kluyveromyces-marxianus->Candida-zeylanoides***	**15.24789698**
**dist_bray**	**copresence**	***Saccharomyces-paradoxus->Streptococcus--***	**0.29653374**
**correl_spearman**	**copresence**	***Leuconostocaceae2->Staphylococcus-equorum***	**0.69792921**
**dist_kullbackleibler**	**copresence**	***Saccharomyces-paradoxus->Streptococcus--***	**1.52466395**
**dist_kullbackleibler**	**copresence**	***Lactobacillus-brevis->Mycoplasma***	**1.1778964**
**dist_bray**	**mutualExclusion**	***Lactobacillus-helveticus->Lactobacillus***	**0.97470171**

**dist_bray**	**mutualExclusion**	***Penicillium-carneum->Penicillium***	**0.93305192**
**correl_pearson**	**mutualExclusion**	***Penicillium->Pseudomonas***	**-0.72711059**
**correl_spearman**	**copresence**	***Lactobacillus-helveticus->Streptococcus--***	**0.83557275**
**dist_kullbackleibler**	**mutualExclusion**	***Hanseniaspora-nectarophila->Lactococcus***	**15.1720763**
**correl_spearman**	**copresence**	***Malassezia-restricta->Hanseniaspora-nectarophila***	**0.8**
**correl_spearman**	**copresence**	***Malassezia-restricta->Lactobacillus-delbrueckii***	**0.80075722**
**correl_spearman**	**copresence**	***Debaryomyces-hansenii->Lactococcus***	**0.80075722**
**correl_pearson**	**mutualExclusion**	***Penicillium-carneum->Malassezia-restricta***	**-0.4610206**
**correl_spearman**	**mutualExclusion**	***Kluyveromyces-marxianus->Candida-zeylanoides***	**-0.64705882**
**dist_bray**	**mutualExclusion**	***Lactococcus->Penicillium-commune***	**0.93959456**
**correl_spearman**	**mutualExclusion**	***Streptococcus--->Leuconostocaceae2***	**-0.51046472**
**correl_pearson**	**copresence**	***Staphylococcus-equorum->Lactobacillus-brevis***	**0.90168506**
**dist_bray**	**copresence**	***Lactobacillus-delbrueckii->Penicillium***	**0.26518425**
**correl_pearson**	**copresence**	***Lactobacillus-delbrueckii->Penicillium***	**0.79710067**
**dist_kullbackleibler**	**copresence**	***Lactobacillus-zeae->Staphylococcus-equorum***	**1.13275599**
**correl_spearman**	**mutualExclusion**	***Penicillium-carneum->Hanseniaspora***	**-0.50460264**

**correl_pearson**	**mutualExclusion**	***Pseudomonas->Hanseniaspora***	**-0.69455355**
**dist_kullbackleibler**	**mutualExclusion**	***Lactobacillus->Penicillium-commune***	**12.20891527**
**dist_bray**	**mutualExclusion**	***Candida-zeylanoides->Lactobacillus-helveticus***	**0.9007962**
**correl_pearson**	**copresence**	***Candida-parapsilosis->Malassezia-restricta***	**0.73818476**
**dist_kullbackleibler**	**mutualExclusion**	***Staphylococcus-equorum->Lactobacillus-helveticus***	**15.2321259**
**dist_bray**	**copresence**	***Penicillium-commune->Penicillium-carneum***	**0.40363456**
**dist_kullbackleibler**	**copresence**	***Lactobacillus-delbrueckii->Penicillium***	**0.81346988**
**correl_spearman**	**copresence**	***Hanseniaspora->Penicillium***	**0.94545455**
**dist_bray**	**copresence**	***Penicillium->Malassezia-restricta***	**0.23374388**
**dist_bray**	**mutualExclusion**	***Lactobacillus->Penicillium-commune***	**0.90437156**
**dist_bray**	**mutualExclusion**	***Lactobacillaceae-->Lactobacillus-helveticus***	**0.94662669**
**correl_pearson**	**mutualExclusion**	***Pseudomonas->Lactobacillus-brevis***	**-0.63784598**
**dist_bray**	**mutualExclusion**	***Staphylococcus-equorum->Lactobacillus-helveticus***	**0.95647934**
**dist_kullbackleibler**	**mutualExclusion**	***Hanseniaspora-nectarophila->Lactobacillus***	**14.63844047**
**correl_pearson**	**mutualExclusion**	***Debaryomyces-hansenii->Penicillium***	**-0.53187761**
**correl_spearman**	**mutualExclusion**	***Hanseniaspora-nectarophila->Penicillium-carneum***	**-0.59312942**

**correl_pearson**	**copresence**	***Lactobacillaceae-->Lactobacillus***	**0.99469198**
**correl_spearman**	**copresence**	***Lactobacillus-zeae->Staphylococcus-equorum***	**0.89843414**
**correl_pearson**	**mutualExclusion**	***Pseudomonas->Lactobacillus-zeae***	**-0.50690995**
**dist_kullbackleibler**	**mutualExclusion**	***Issatchenkia-orientalis->Candida-zeylanoides***	**16.13834303**
**dist_bray**	**copresence**	***Lactobacillaceae-->Lactobacillus***	**0.13584518**
**correl_spearman**	**copresence**	***Lactobacillaceae-->Lactococcus***	**0.7572378**
**correl_spearman**	**mutualExclusion**	***Staphylococcus-equorum->Lactobacillus-helveticus***	**-0.67280352**
**correl_pearson**	**mutualExclusion**	***Malassezia-restricta->Debaryomyces-hansenii***	**-0.63627649**
**correl_pearson**	**copresence**	***Streptococcus--->Pseudomonas***	**0.78545768**
**dist_kullbackleibler**	**copresence**	***Debaryomyces-hansenii->Lactobacillaceae-***	**1.60899274**
**correl_pearson**	**copresence**	***Debaryomyces-hansenii->Lactococcus***	**0.70877117**
**correl_spearman**	**copresence**	***Lactobacillus-zeae->Lactobacillus***	**0.70501451**
**dist_bray**	**mutualExclusion**	***Lactococcus->Malassezia-restricta***	**0.92584245**
**dist_bray**	**copresence**	***Malassezia-restricta->Hanseniaspora-nectarophila***	**0.28278045**
**dist_kullbackleibler**	**copresence**	***Lactobacillaceae-->Lactococcus***	**1.17083299**
**correl_spearman**	**copresence**	***Hanseniaspora->Lactobacillus-delbrueckii***	**0.94001934**
**dist_bray**	**mutualExclusion**	***Hanseniaspora-nectarophila->Lactococcus***	**0.92584245**
**dist_bray**	**mutualExclusion**	***Lactobacillus-brevis->Penicillium-carneum***	**0.94318417**

dist_kullbackleibler	copresence	*Penicillium->Malassezia-restricta*	0.38456587
correl_spearman	mutualExclusion	*Lactococcus->Lactobacillus-delbrueckii*	-0.7572378
dist_kullbackleibler	mutualExclusion	*Hanseniaspora-nectarophila->Lactobacillaceae-*	12.23547414
dist_bray	mutualExclusion	*Issatchenkia-orientalis->Candida-zeylanoides*	0.92223915
dist_kullbackleibler	copresence	*Debaryomyces-hansenii->Streptococcus--*	0.47186974
dist_bray	mutualExclusion	*Lactobacillus-helveticus->Issatchenkia-orientalis*	0.91678767
correl_pearson	mutualExclusion	*Lactococcus->Lactobacillus-delbrueckii*	-0.47176739
dist_bray	copresence	*Leuconostocaceae2->Staphylococcus-equorum*	0.20033033
dist_bray	copresence	*Mycoplasma->Hanseniaspora*	0.40813366
correl_pearson	mutualExclusion	*Lactobacillus-brevis->Streptococcus--*	-0.75268694
dist_bray	mutualExclusion	*Lactococcus->Hanseniaspora*	0.93546813
correl_spearman	mutualExclusion	*Lactobacillus->Candida-parapsilosis*	-0.67280352
dist_bray	mutualExclusion	*Lactobacillus-brevis->Lactobacillus-helveticus*	0.91029962
dist_kullbackleibler	mutualExclusion	*Hanseniaspora->Lactobacillus*	13.14278744
dist_bray	copresence	*Streptococcus--->Pseudomonas*	0.27395854
dist_kullbackleibler	copresence	*Staphylococcus-equorum->Lactobacillaceae-*	1.3249554
dist_bray	mutualExclusion	*Penicillium->Lactococcus*	0.93049025
dist_bray	copresence	*Lactobacillus-delbrueckii->Hanseniaspora-nectarophila*	0.32669665

dist_kullbackleibler	mutualExclusion	*Lactobacillus-brevis->Lactobacillus-helveticus*	12.67230528
correl_pearson	copresence	*Lactobacillaceae-->Lactococcus*	0.7590011
correl_pearson	mutualExclusion	*Pseudomonas->Mycoplasma*	-0.46181727
correl_pearson	mutualExclusion	*Streptococcus--->Leuconostocaceae2*	-0.78614749
correl_spearman	mutualExclusion	*Debaryomyces-hansenii->Penicillium*	-0.5048252
correl_spearman	mutualExclusion	*Penicillium->Lactococcus*	-0.55909091
correl_pearson	copresence	*Penicillium-commune->Penicillium-carneum*	0.78975403
correl_pearson	copresence	*Hanseniaspora->Lactobacillus-delbrueckii*	0.70263437
dist_kullbackleibler	mutualExclusion	*Penicillium->Lactococcus*	12.35634664
correl_spearman	copresence	*Lactobacillaceae-->Lactobacillus*	0.82686887
dist_bray	copresence	*Staphylococcus-equorum->Lactobacillus-brevis*	0.24284369
dist_bray	copresence	*Hanseniaspora->Penicillium*	0.22878503
correl_spearman	mutualExclusion	*Hanseniaspora-nectarophila->Lactobacillus*	-0.49545455
correl_pearson	mutualExclusion	*Lactobacillus-helveticus->Lactobacillus-zeae*	-0.45760967
correl_pearson	mutualExclusion	*Hanseniaspora-nectarophila->Lactococcus*	-0.54664906
dist_kullbackleibler	mutualExclusion	*Hanseniaspora-nectarophila->Penicillium-commune*	15.71610129
dist_kullbackleibler	copresence	*Lactobacillus-delbrueckii->Hanseniaspora-nectarophila*	1.16825564
dist_bray	mutualExclusion	*Penicillium-commune->Issatchenkia-orientalis*	0.91633967

**correl_pearson**	**copresence**	***Lactococcus->Lactobacillus***	**0.74293151**
**dist_bray**	**copresence**	***Debaryomyces-hansenii->Pseudomonas***	**0.40732754**
**correl_pearson**	**mutualExclusion**	***Lactococcus->Malassezia-restricta***	**-0.50271234**
**dist_kullbackleibler**	**copresence**	***Streptococcus--->Pseudomonas***	**1.28903766**
**correl_pearson**	**mutualExclusion**	***Pseudomonas->Leuconostocaceae2***	**-0.63005015**
**correl_spearman**	**copresence**	***Lactobacillus-delbrueckii->Hanseniaspora-nectarophila***	**0.81816498**
**correl_pearson**	**mutualExclusion**	***Penicillium->Lactococcus***	**-0.4953524**
**correl_spearman**	**copresence**	***Mycoplasma->Kluyveromyces-marxianus***	**0.76601987**
**correl_pearson**	**mutualExclusion**	***Pseudomonas->Lactobacillus-delbrueckii***	**-0.5631326**
**dist_bray**	**mutualExclusion**	***Penicillium-carneum->Malassezia-restricta***	**0.9048189**
**correl_spearman**	**copresence**	***Penicillium->Malassezia-restricta***	**0.87272727**
**correl_spearman**	**mutualExclusion**	***Lactobacillus-helveticus->Lactobacillus***	**-0.52272727**
**correl_pearson**	**mutualExclusion**	***Staphylococcus-equorum->Streptococcus--***	**-0.77002716**
**correl_pearson**	**copresence**	***Lactobacillus-zeae->Leuconostocaceae2***	**0.71901195**
**dist_kullbackleibler**	**mutualExclusion**	***Lactococcus->Hanseniaspora***	**14.96376663**
**correl_pearson**	**copresence**	***Lactobacillus-helveticus->Streptococcus--***	**0.69098907**
**correl_spearman**	**mutualExclusion**	***Lactobacillaceae--->Lactobacillus-delbrueckii***	**-0.51666667**
**dist_bray**	**mutualExclusion**	***Candida-zeylanoides->Lactobacillus-brevis***	**0.89879477**

**dist_bray**	**copresence**	***Mycoplasma->Leuconostocaceae2***	**0.19321637**
**correl_spearman**	**mutualExclusion**	***Staphylococcus-equorum->Streptococcus--***	**-0.66111192**
**dist_kullbackleibler**	**mutualExclusion**	***Lactobacillus-helveticus->Hanseniaspora***	**12.820631**
**correl_spearman**	**mutualExclusion**	***Penicillium->Pseudomonas***	**-0.51345532**
**correl_spearman**	**mutualExclusion**	***Lactobacillus-brevis->Streptococcus--***	**-0.53093685**
**correl_spearman**	**copresence**	***Hanseniaspora->Malassezia-restricta***	**0.85454545**
**dist_bray**	**copresence**	***Leuconostocaceae2->Lactobacillus-brevis***	**0.08027394**
**dist_kullbackleibler**	**mutualExclusion**	***Lactococcus->Penicillium-commune***	**15.2962946**
**correl_pearson**	**mutualExclusion**	***Hanseniaspora-nectarophila->Penicillium-carneum***	**-0.47057452**
**correl_spearman**	**copresence**	***Lactobacillus-zeae->Leuconostocaceae2***	**0.68619848**
**correl_spearman**	**copresence**	***Staphylococcus-equorum->Lactobacillaceae-***	**0.83062779**
**correl_spearman**	**copresence**	***Debaryomyces-hansenii->Lactobacillaceae-***	**0.66666667**
**dist_kullbackleibler**	**copresence**	***Staphylococcus-equorum->Lactobacillus-brevis***	**0.94904241**
**correl_pearson**	**copresence**	***Lactobacillus-brevis->Mycoplasma***	**0.9526956**
**dist_kullbackleibler**	**mutualExclusion**	***Penicillium-carneum->Hanseniaspora***	**14.57738619**
**dist_bray**	**mutualExclusion**	***Lactobacillus->Candida-parapsilosis***	**0.925499**
**correl_pearson**	**copresence**	***Debaryomyces-hansenii->Lactobacillaceae-***	**0.72261524**
**dist_bray**	**copresence**	***Lactobacillus-zeae->Staphylococcus-equorum***	**0.35313418**

**correl_spearman**	**copresence**	***Staphylococcus-equorum->Lactobacillus***	**0.69936155**
**correl_spearman**	**mutualExclusion**	***Malassezia-restricta->Debaryomyces-hansenii***	**-0.62667956**
**correl_spearman**	**mutualExclusion**	***Lactococcus->Hanseniaspora***	**-0.62272727**
**dist_kullbackleibler**	**mutualExclusion**	***Mycoplasma->Candida-zeylanoides***	**12.52308384**
**correl_pearson**	**copresence**	***Staphylococcus-equorum->Mycoplasma***	**0.86494591**
**dist_bray**	**mutualExclusion**	***Penicillium-carneum->Hanseniaspora***	**0.9380298**
**correl_pearson**	**mutualExclusion**	***Debaryomyces-hansenii->Hanseniaspora-nectarophila***	**-0.60008835**
**correl_spearman**	**mutualExclusion**	***Debaryomyces-hansenii->Lactobacillus-delbrueckii***	**-0.58333333**
**dist_kullbackleibler**	**mutualExclusion**	***Leuconostocaceae2->Lactobacillus-helveticus***	**13.47126651**
**dist_kullbackleibler**	**copresence**	***Hanseniaspora->Lactobacillus-delbrueckii***	**1.09252785**
**correl_pearson**	**copresence**	***Lactobacillus->Debaryomyces-hansenii***	**0.71821462**
**correl_spearman**	**copresence**	***Lactobacillaceae-->Lactobacillus-zeae***	**0.83333333**
**correl_pearson**	**copresence**	***Lactobacillus-delbrueckii->Hanseniaspora-nectarophila***	**0.69629743**
**dist_kullbackleibler**	**mutualExclusion**	***Penicillium-commune->Candida-zeylanoides***	**13.31456245**
**dist_bray**	**copresence**	***Debaryomyces-hansenii->Streptococcus--***	**0.25492358**
**dist_bray**	**mutualExclusion**	***Lactobacillus-helveticus->Lactobacillus-zeae***	**0.97635856**

**dist_kullbackleibler**	**mutualExclusion**	***Hanseniaspora->Penicillium-commune***	**13.84412247**
**correl_spearman**	**mutualExclusion**	***Issatchenkia-orientalis->Candida-zeylanoides***	**-0.6302521**
**correl_pearson**	**copresence**	***Leuconostocaceae2->Lactobacillus-brevis***	**0.98890923**
**dist_bray**	**mutualExclusion**	***Leuconostocaceae2->Lactobacillus-helveticus***	**0.96143165**
**dist_kullbackleibler**	**copresence**	***Penicillium->Hanseniaspora-nectarophila***	**0.69771379**
**dist_kullbackleibler**	**copresence**	***Lactobacillaceae-->Lactobacillus***	**0.27475626**
**correl_pearson**	**mutualExclusion**	***Pseudomonas->Staphylococcus-equorum***	**-0.54228474**
**correl_pearson**	**mutualExclusion**	***Staphylococcus-equorum->Lactobacillus-helveticus***	**-0.4582689**
**dist_kullbackleibler**	**copresence**	***Malassezia-restricta->Hanseniaspora-nectarophila***	**0.66572075**
**correl_pearson**	**mutualExclusion**	***Streptococcus--->Lactobacillus-zeae***	**-0.66347592**
**correl_spearman**	**copresence**	***Penicillium->Hanseniaspora-nectarophila***	**0.81818182**
**correl_pearson**	**mutualExclusion**	***Hanseniaspora->Debaryomyces-hansenii***	**-0.50052222**
**correl_spearman**	**mutualExclusion**	***Debaryomyces-hansenii->Hanseniaspora-nectarophila***	**-0.57445626**
**correl_pearson**	**mutualExclusion**	***Lactococcus->Hanseniaspora***	**-0.51475312**
**dist_bray**	**copresence**	***Lactobacillus-zeae->Leuconostocaceae2***	**0.32890764**
**correl_spearman**	**copresence**	***Lactobacillus-delbrueckii->Penicillium***	**0.94001934**
**correl_pearson**	**copresence**	***Lactobacillus-brevis->Lactobacillus-zeae***	**0.71033026**
**dist_kullbackleibler**	**mutualExclusion**	***Lactobacillus-helveticus->Lactobacillus***	**14.93181664**

**correl_pearson**	**mutualExclusion**	***Streptococcus--->Mycoplasma***	**-0.64873346**
**dist_bray**	**copresence**	***Penicillium->Hanseniaspora-nectarophila***	**0.29338074**
**dist_kullbackleibler**	**mutualExclusion**	***Penicillium->Lactobacillus-helveticus***	**12.10577452**
**dist_kullbackleibler**	**copresence**	***Malassezia-restricta->Lactobacillus-delbrueckii***	**1.34979225**
**dist_kullbackleibler**	**copresence**	***Leuconostocaceae2->Staphylococcus-equorum***	**0.54201949**
**dist_bray**	**copresence**	***Lactobacillus-brevis->Lactobacillus-zeae***	**0.33965697**
**correl_pearson**	**copresence**	***Mycoplasma->Leuconostocaceae2***	**0.93914167**
**dist_kullbackleibler**	**mutualExclusion**	***Hanseniaspora-nectarophila->Penicillium-carneum***	**15.84629174**
**correl_spearman**	**mutualExclusion**	***Pseudomonas->Hanseniaspora***	**-0.51345532**
**dist_kullbackleibler**	**mutualExclusion**	***Penicillium-carneum->Penicillium***	**13.02761942**
**dist_kullbackleibler**	**copresence**	***Hanseniaspora->Malassezia-restricta***	**0.72574822**
**correl_pearson**	**copresence**	***Penicillium->Malassezia-restricta***	**0.74581046**
**correl_pearson**	**copresence**	***Hanseniaspora-nectarophila->Hanseniaspora***	**0.76288228**
**dist_bray**	**copresence**	***Hanseniaspora->Lactobacillus-delbrueckii***	**0.30953415**
**correl_spearman**	**copresence**	***Leuconostocaceae2->Issatchenkia-orientalis***	**0.70168067**
**correl_spearman**	**mutualExclusion**	***Lactococcus->Malassezia-restricta***	**-0.49545455**
**dist_bray**	**copresence**	***Lactobacillus-brevis->Mycoplasma***	**0.14326704**
**dist_bray**	**copresence**	***Lactobacillaceae--->Lactococcus***	**0.377428**

**dist_kullbackleibler**	**mutualExclusion**	***Lactobacillaceae-->Penicillium-commune***	**12.11443655**
**dist_bray**	**copresence**	***Mycoplasma->Lactobacillus-zeae***	**0.36781285**
**correl_spearman**	**copresence**	***Hanseniaspora-nectarophila->Hanseniaspora***	**0.83636364**
**dist_bray**	**copresence**	***Staphylococcus-equorum->Mycoplasma***	**0.2631117**
**correl_spearman**	**copresence**	***Streptococcus--->Pseudomonas***	**0.76282144**
**dist_bray**	**mutualExclusion**	***Leuconostocaceae2->Penicillium-carneum***	**0.93851296**
**dist_kullbackleibler**	**copresence**	***Hanseniaspora-nectarophila->Hanseniaspora***	**0.39063642**
**dist_kullbackleibler**	**copresence**	***Streptococcus--->Lactobacillus-delbrueckii***	**1.56186047**
**dist_kullbackleibler**	**copresence**	***Debaryomyces-hansenii->Pseudomonas***	**1.44468302**
**correl_spearman**	**mutualExclusion**	***Penicillium-carneum->Penicillium***	**-0.522308**
**dist_kullbackleibler**	**copresence**	***Leuconostocaceae2->Lactobacillus-brevis***	**0.60795596**
**dist_kullbackleibler**	**copresence**	***Mycoplasma->Leuconostocaceae2***	**1.38300121**
**correl_spearman**	**mutualExclusion**	***Mycoplasma->Candida-zeylanoides***	**-0.51919124**
**correl_pearson**	**copresence**	***Hanseniaspora->Penicillium***	**0.75206557**
**dist_kullbackleibler**	**mutualExclusion**	***Lactococcus->Lactobacillus-helveticus***	**12.20781254**
**dist_bray**	**mutualExclusion**	***Penicillium-commune->Candida-zeylanoides***	**0.92532065**
**correl_pearson**	**copresence**	***Lactobacillus-zeae->Staphylococcus-equorum***	**0.67472595**
**dist_bray**	**copresence**	***Hanseniaspora-nectarophila->Hanseniaspora***	**0.25590026**

**dist_bray**	**mutualExclusion**	***Hanseniaspora-nectarophila->Penicillium-carneum***	**0.90338184**
**dist_kullbackleibler**	**copresence**	***Lactobacillus-zeae->Leuconostocaceae2***	**1.58730982**
**correl_pearson**	**copresence**	***Leuconostocaceae2->Staphylococcus-equorum***	**0.92946669**
**correl_spearman**	**mutualExclusion**	***Lactobacillus->Streptococcus--***	**-0.62667956**
**dist_kullbackleibler**	**copresence**	***Hanseniaspora->Penicillium***	**0.33241245**
